# *In vitro* and *in silico* Models to Study Mosquito-Borne Flavivirus Neuropathogenesis, Prevention, and Treatment

**DOI:** 10.3389/fcimb.2019.00223

**Published:** 2019-07-09

**Authors:** Megan Chesnut, Laura S. Muñoz, Georgina Harris, Dana Freeman, Lucio Gama, Carlos A. Pardo, David Pamies

**Affiliations:** ^1^Center for Alternatives to Animal Testing, Johns Hopkins Bloomberg School of Public Health, Baltimore, MD, United States; ^2^Division of Neuroimmunology, Department of Neurology, Johns Hopkins University School of Medicine, Baltimore, MD, United States; ^3^Neuroviruses Emerging in the Americas Study, Johns Hopkins University School of Medicine, Baltimore, MD, United States; ^4^Department of Environmental Health and Engineering, Johns Hopkins Bloomberg School of Public Health, Baltimore, MD, United States; ^5^Department of Molecular and Comparative Pathobiology, Johns Hopkins University School of Medicine, Baltimore, MD, United States; ^6^Vaccine Research Center, National Institute of Allergy and Infectious Diseases, Bethesda, MD, United States; ^7^Department of Physiology, University of Lausanne, Lausanne, Switzerland

**Keywords:** mosquito-borne flavivirus, flaviviridae, Zika virus (ZIKV), West Nile virus (WNV), Dengue virus (DENV), neuropathogenesis, *in vitro*, *in silico*

## Abstract

Mosquito-borne flaviviruses can cause disease in the nervous system, resulting in a significant burden of morbidity and mortality. Disease models are necessary to understand neuropathogenesis and identify potential therapeutics and vaccines. Non-human primates have been used extensively but present major challenges. Advances have also been made toward the development of humanized mouse models, but these models still do not fully represent human pathophysiology. Recent developments in stem cell technology and cell culture techniques have allowed the development of more physiologically relevant human cell-based models. *In silico* modeling has also allowed researchers to identify and predict transmission patterns and discover potential vaccine and therapeutic candidates. This review summarizes the research on *in vitro* and *in silico* models used to study three mosquito-borne flaviviruses that cause neurological disease in humans: West Nile, Dengue, and Zika. We also propose a roadmap for 21st century research on mosquito-borne flavivirus neuropathogenesis, prevention, and treatment.

## Introduction

Mosquito-borne flaviviruses (MBFs) are geographically widespread viral infections and a significant cause of morbidity and mortality worldwide (Huang et al., 2014). Neurovirulent MBFs that have recently emerged in the Western world, such as Dengue virus (DENV), Zika virus (ZIKV), and West Nile virus (WNV), deserve attention due to the unexpected high burden of associated neurological disease observed in affected regions (Sips et al., 2012; Muñoz et al., 2017). WNV was endemic in some regions in Europe, Asia and Africa where it caused sporadic outbreaks (Sambri et al., 2013). It emerged for the first time in America in 1999 and since has become the most widespread flavivirus that causes neurological disease globally (Chancey et al., 2015). One in 150 individuals infected by WNV experience a neurological disease characterized by encephalitis, meningoencephalitis, meningitis, and flaccid paralysis (Tsai et al., 1998; Chowers et al., 2001; Mostashari et al., 2001). Mortality due to WNV encephalitis ranges between 10 and 17% and increases with age and immunosuppression state (Petersen et al., 2013). DENV is the second most common mosquito-borne infection and is found in tropical and subtropical regions (Caraballo and King, 2014). Neurological symptoms are typically observed in 4–9% of infected subjects (Solomon et al., 2000; Pancharoen and Thisyakorn, 2001; Sahu et al., 2014), though it has been documented as high as 21% of cases in some reports (Domingues et al., 2008). ZIKV is the most recent emerging MBF in the Americas. After its arrival in 2015, an increased incidence of microcephaly in newborns and Guillain-Barré syndrome (GBS) in adults infected with ZIKV was reported (do Rosário et al., 2016). Today, it is known that ZIKV causes fetal brain malformations and triggers GBS (Beckham et al., 2016).

WNV, DENV, and ZIKV RNA have been found in the cerebrospinal fluid of infected patients with neurological symptoms (Huang et al., 2002; Carteaux et al., 2016) but the mechanism by which these viruses elicit a neurological effect remains unclear. Studies on the tropism of MBF and the associated histopathological changes following natural human infection have been reported. However, published results from human studies are limited and highly descriptive because they do not reflect the evolving dynamic between the host and the pathogen, which is crucial to understand the pathophysiology of these infections (Bearcroft, 1956; Armah et al., 2007). Because of the limited number of preclinical *in vivo* studies demonstrating efficacy, clinical trials for MBF drug candidates are scarce. Several candidates of a human WNV vaccine have undergone pre-clinical testing mainly in mice, horses, and NHPs (Amanna and Slifka, 2014), but none has been licensed for use in humans. Clinical trials for ZIKV vaccines are still in the early stages. Only one MBF vaccine, CYD-TDV or “Dengvaxia,” a recombinant, chimeric, live attenuated tetravalent vaccine for DENV has been licensed. The manufacturing and licensing of antiviral drugs has also been challenging due to a lack of suitable models to evaluate novel agents. Supportive treatment is the current standard of care and most prevention strategies are focused on decreasing the exposure to the arthropod vectors responsible for the transmission of MBFs. The development of experimental models of neuropathogenesis following WNF and ZIKV infection has thus been a central focus in preclinical vaccine research and antiviral drug development of this virus (Chan et al., 2015). However, neuropathgogenesis of DENV is generally less well studies and not a focus of vaccine or anti-viral research.

Small rodents, birds, medium-sized mammals, and non-human primates (NHPs) have been used in MBF research (Kimura et al., 2010; Goodfellow et al., 2016; Maximova et al., 2016). NHPs have traditionally been used to study human viruses because much of the host-specific pathophysiology is conserved between primates and humans. Protective efficacy of novel vaccines for MBFs has been demonstrated in primates for DENV (Guirakhoo et al., 2004), WNV (Petersen et al., 2013), and ZIKV (Abbink et al., 2016), but their efficacy in humans still needs to be corroborated (Torresi et al., 2017; Vannice et al., 2019). Primates also present maintenance challenges due to high cost and the strict requirements of high-containment biosafety facilities.

Mouse models are a more cost-effective and practical alternative to NHP models. The use of mouse models has resulted in a robust body of knowledge in MBF research, and this has been reviewed elsewhere (Clark et al., 2012). However, two main factors are recognized to influence the susceptibility of mice to MBF infection that limit applicability to humans: (1) immunocompetent wild-type mouse strains have been demonstrated to favor resistance to disease after peripheral inoculation with DENV and ZIKV (Clark et al., 2012; Zompi and Harris, 2012), so immunocompromised mice (Dhole et al., 2016; Oh et al., 2017; Kuszpit et al., 2018), humanized mice (An et al., 1999), and mouse-adapted viral strains (Amorim et al., 2012; Daniels et al., 2017) are used and (2) in mice, MBF infection via mosquito biting, the natural route of infection in humans, results in a lack of severe clinical manifestations (Styer et al., 2011; Cox et al., 2012; Zompi and Harris, 2012; Moser et al., 2016), so alternative routes of infection, such as subcutaneous (Smith et al., 2017), intraocular (van den Pol et al., 2017), intraperitoneal, intravenous (Xavier-Neto et al., 2017) and mucosal (Yockey et al., 2016), have been used. Among those routes, intracranial inoculation has been used most, but it does not elicit the early peripheral immune response that occurs in humans (Kimura et al., 2010; Shao et al., 2017). For more information about animal models, see Supplementary Tables 1–5.

*In vitro* human cell-based and *in silico* models have emerged as useful tools in MBF research with the goal of clarifying viral neurotropism and neuropathogenesis, accelerating the process of antiviral drug and vaccine development, and understanding transmission patterns. Besides, there are other flaviviruses able to cause neurological diseases (such as Japanese encephalitis and tick-borne encephalitis virus), and due to the size of the review search, we have focused this review on 3 of the main flavivirus with neurological pathology as an example of the flavivirus research. This review aims to summarize the *in vitro* and *in silico* research on DENV, WNV, and ZIKV and to define a roadmap for 21st century biomedical research on MBF neuropathogenesis, prevention, and treatment.

### Vaccine Research

There is a call to reduce the global burden of neurological disease caused by flavivirus. DENV continues its steady expansion and constitutes the most common mosquito-borne viral disease worldwide. WNV is the most widespread flavivirus causing neurological disease in the world and exemplifies the potential transmission of flaviviruses outside tropical regions. ZIKV recently caused an unpredicted epidemic in the Americas is responsible for thousands of cases of microcephaly in newborns and Guillain-Barre syndrome in adults. Different strategies have been studied to limit the exposure to these infections and eradicate the occurrence of neurological involvement. Vector control has been explored with limited results. Therefore, vaccination is the mainstay intervention to prevent flavivirus infection.

Though an efficient WNV vaccine for humans is not available, vaccination of equine population has been a successful strategy to immunize control human transmission (Ng et al., 2003; El Garch et al., 2008). This is possible because WNV primary reservoirs are birds and horses, while humans are dead-end hosts for the virus. WNV live attenuated vaccines are the most advanced and had efficiently passed phase II clinical trials (Biedenbender et al., 2011). Subunit and DNA vaccines encoding E and prM proteins are also being developed. DNA vaccines have passed phase I clinical trials, showing safety and immunogenicity (Martin et al., 2007; Ledgerwood et al., 2011).

The development of a DENV vaccine has been a complex task to accomplish for different reasons. First, there is no an adequate animal model that recapitulates human DENV infection. Second, DENV has four antigenically different serotypes, each with intrinsic genetic variation. The natural infection with one serotype confers long term immunity against the infecting serotype, but the humoral response fails to neutralize the other serotypes. Third, DENV heterotypic infections have been associated with an increased likelihood of severe clinical manifestations possibly attributed to an immune enhancement of the infection driven by cross-reactive non-neutralizing antibodies (Collins and Metz, 2017). Due to these concerns the development of a DENV tetravalent vaccine has been the focus over the 21st century; this vaccine should elicit a rise long-lasting protective immunity against all the serotypes. Several phase I and II clinical trials have been completed using monovalent or tetravalent live attenuated (Edelman et al., 2003; Sun et al., 2009; Wright et al., 2009; Capeding et al., 2011; Poo et al., 2011; Leo et al., 2012; Sabchareon et al., 2012; Durbin et al., 2013; Villar et al., 2013; Watanaveeradej et al., 2014; Bauer et al., 2015; George et al., 2015; Dubey et al., 2016; Whitehead et al., 2017), DNA (Beckett et al., 2011), chimeric (Guirakhoo et al., 2006), and purified inactivated vaccines (Martinez et al., 2015). CYD-TDV, a recombinant, chimeric, live attenuated vaccine is the only one licensed for individuals between 9 and 45 years in more than 10 endemic countries and has completed phase III clinical trials in Asia and Latin America (Capeding et al., 2014; Villar et al., 2015).

There are no approved vaccines against ZIKV infection. Tebas et al. conducted a phase I clinical trial to evaluate the safety and efficacy of the DNA vaccine (GLS-5700) which encodes the premembrane and envelope proteins in healthy individuals. The vaccine elicited anti-ZIKV neutralizing antibodies in 62% of the samples on a cellular assay (Tebas et al., 2017). There are some phase I and II clinical trials on ZIKV vaccine. However, the preliminary results will be available a couple of years from now.

Achieving sub-neutralizing levels of antibodies, on the contrary, might increase the risk of an immunologically mediated severe disease. However, other vaccine platforms have also been tested since the early 90s.

The most recent clinical trial published in 2017, demonstrated safety of vaccination in individuals previously exposed to flavivirus. The TV003 vaccine was not associated with severe disease in this group (Whitehead et al., 2017). Immunogenicity of the tetravalent CYD-TDV vaccine was demonstrated in naïve and seropositive patients to DENV infection in India in 2016 (Dubey et al., 2016). In 2015, the re-derived, live attenuated, tetravalent dengue virus (TDEN) vaccine also demonstrated to be safety and immunogenic for in individual ranging from 1 to 50 years of age independently of a prior exposure to the infection in Puerto Rico (Bauer et al., 2015). The same year, the DENV-1 purified inactivated vaccine was tested in flavivirus-naïve healthy adults in the United States, in which humoral response was effectively detected (Martinez et al., 2015). In 2015, the live attenuated tetravalent vaccine TDV demonstrated to be safe and induce anti-DENV neutralizing antibodies in flavivirus-naïve adults in the United States (George et al., 2015). In 2016, the WHO recommended the use of Dengvaxia, a live attenuated tetravalent vaccine, in regions with 70% of DENV seroprevalence. To date Dengvaxia is licensed in 11 countries for people age 9–45 years.

Phase III trials have suggested high rate of protection among partial immune individuals, but high rates of hospitalizations in small children who were seronegative prior to the vaccination (Aguiar et al., 2016).

### Vaccination to Veterinary Population

Phase I clinical trials have been published to assess 2 DNA vaccines encoding the pre-membrane and the envelope glycoprotein of the NY99 strain of WNV, in 2007 and 2011 (Martin et al., 2007; Ledgerwood et al., 2011). Both studies demonstrated safety and efficacy in inducing neutralizing antibodies in individuals who completed 2 or 3 doses of vaccination.

DENV vaccine development has been limited by the lack of animal models recapitulating the infection and the risks attributed to past exposure to DENV infections, other flaviviruses or similar vaccines.

### Drugs

Human clinical trials have been used to assess no pharmacological strategies to prevent flavivirus infection. There are no current treatments available for DENV infection. In 2016, a clinical trial testing celgosivir, an endoplasmic reticulum alpha glucosidase inhibitor; failed to demonstrate antiviral efficacy (lower viremia) in Asia (Sung et al., 2016). Similarly, in the same year, lovastatin showed no power to address efficacy in Vietnam (Whitehorn et al., 2016). A protocol of fluid resuscitation with hypertonic sodium lactate showed similar efficacy compared to the Ringer Lactate in patients in Indonesia with dengue shock syndrome, based on rate of recovery (Somasetia et al., 2014).

## Literature Review Strategy

A PubMed search was conducted to determine the distribution of articles between *in vitro* and *in silico* studies within the field of MBF research (Table 1).

**Table 1:** PubMed search query to identify *in vitro* and *in silico* studies.

Flaviviridae, flavivirus, flaviviruses, flaviviral	AND	Mosquito-borne, mosquito borne, arbovirus, arboviruses		Organoid, organoids, on-a-chip, spheroid, spheroids, neurosphere, neurospheres, organotypic culture, organotypic cultures, microphysiological system, microphysiological systems		
OR			AND	OR		
Zika, ZIKV, West Nile, WNV, Dengue, DENV				QSAR, molecular mechanism, molecular mechanisms, systems biology, pathway, pathways, model, models, modeling	AND	*in silico*, computational, *in vitro* techniques [mesh], *in vitro*, cell culture, cultured cells, cell-based

Keywords separated by commas (Table 1) were combined with the Boolean operator “OR” and the term: “AND(“2000/01/01”[PDAT]: “2017/10/31”[PDAT])” was added to the end of the query to exclude studies conducted prior to the year 2000.

**Figure 1:**
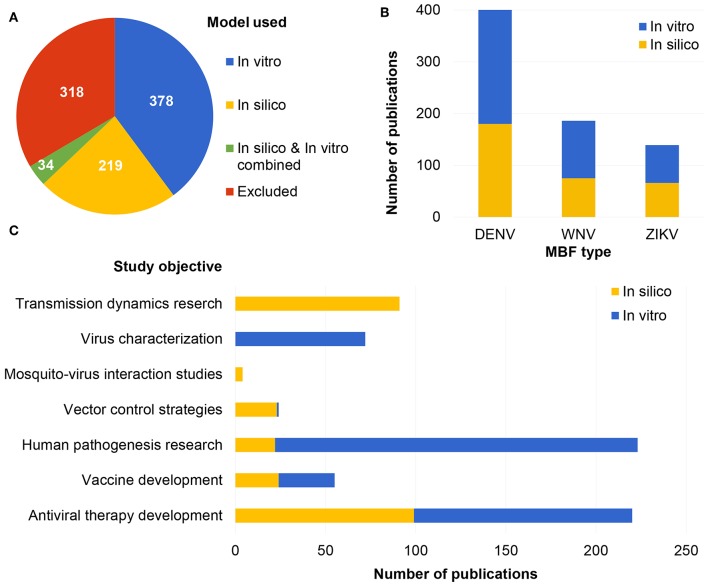
Number of articles included and excluded with corresponding label for type of model used **(A)**, MBFs represented **(B)**, and study objectives **(C)** in the *in vitro* and *in silico* literature.

The search yielded 949 results in PubMed. Article titles, authors, keywords, and abstracts were imported into Sysrev.us (Insilica LLC) and each abstract was reviewed and labeled independently by two individuals. Abstract labels included the following: flavivirus (yes or no), flavivirus type (DENV, WNV, ZIKV, or other), study type (*in vivo, in vitro*, or *in silico*), model species (human, mouse, primate, or other), organ model (brain, biological barrier, immune system, or other), study objective (antiviral therapy development, vaccine development, human pathogenesis, transmission dynamics, vector control, virus characterization, and mosquito-virus interactions), and overall include or exclude along with the option to add notes with a reason for excluding the article (Figure 1). Articles were excluded from the review for the following reasons: article is unavailable, article is not original research (review, opinion, editorial, letter, or comment), article is not in English, study does not pertain to DENV, WNV, or ZIKV, study exclusively uses *in vivo* methods (animal or human), or study is focused on diagnostics or detection methods or the economic burden of disease. After each abstract was labeled by at least two individuals, article information with associated labels was exported as a CSV file from SysRev.us (Insilica LLC). Overall, 318 of papers were excluded from the review. Of the remaining included articles, most (378) were *in vitro* studie followed by *in silico* studies (219) with less (34) studies which combine *in silico* and *in vitro* methods (Figure 1).

## *In vitro* Studies

Due to a need for more human relevant models to study the physiology and mechanisms of MBF infection, research on *in vitro* methods has increased in the last decade. MBF research presents a series of unique challenges, such as human-specific pathology and a lack of adequate animal models. Thus, *in vitro* methods represent a valuable resource in this field. Research on *in vitro* models is also motivated by the search for more cost-effective drug screening methods that could be used to more efficiently screen compounds in drug development stages, with the added benefit of eliminating many of the ethical problems associated with the use of animals for research. *In vitro* methods still have limitations, such as lack of exhaustive reporting, low statistical power, and variability in reagents and protocols, and this has been summarized elsewhere along with Guidance for Good Cell Culture Practice (GCCP) for *in vitro* research (Coecke et al., 2005; Pamies and Hartung, 2017).

Many *in vitro* studies were focused on pathogenesis research (201/412 studies) (Figure 1C). For example, *in vitro* models have been used to study the effects of MBF on early stages of embryonic differentiation (Liang et al., 2016; Gabriel et al., 2017), viral receptors (Tsai et al., 2009; Qian et al., 2011; Dang et al., 2016), the interactions of different cell types during pathogenesis (Diniz et al., 2006; Verma et al., 2009; Bramley et al., 2017), and the mechanism by which the blood brain barrier (BBB) becomes compromised (Roe et al., 2014). *In vitro* methods have also proven useful in drug screening (Allen et al., 2008; Astashkina et al., 2012) and have potential to aid in the selection of drug candidates for future phases of drug development (Xu et al., 2016; Zhou et al., 2017). Recently, a high-content screening platform to study possible antiviral candidates for ZIKV was developed, in which human neural progenitor cells (NPCs) were treated, following ZIKV infection, with over 1,000 US FDA approved drugs and drug candidates (Zhou et al., 2017). Treatments were validated with more complex systems, such as human fetal-like forebrain organoid models and animal studies (Zhou et al., 2017), which demonstrates that *in vitro* methods can aid in selection of drug candidates prior to the use of more complex methods, saving resources and time.

The majority (270/412 studies) of reviewed *in vitro* studies focused on DENV, followed by WNV (111/412 studies) and ZIKV (73/412 studies) (Figure 1B). DENV is considered the most common mosquito-borne infection after malaria; therefore, it was expected that DENV would be the most studied MBF in the *in vitro* literature. There were fewer studies on ZIKV, which is understandable, because ZIKV outbreaks have only raised concern in recent years.

**Figure 2:**
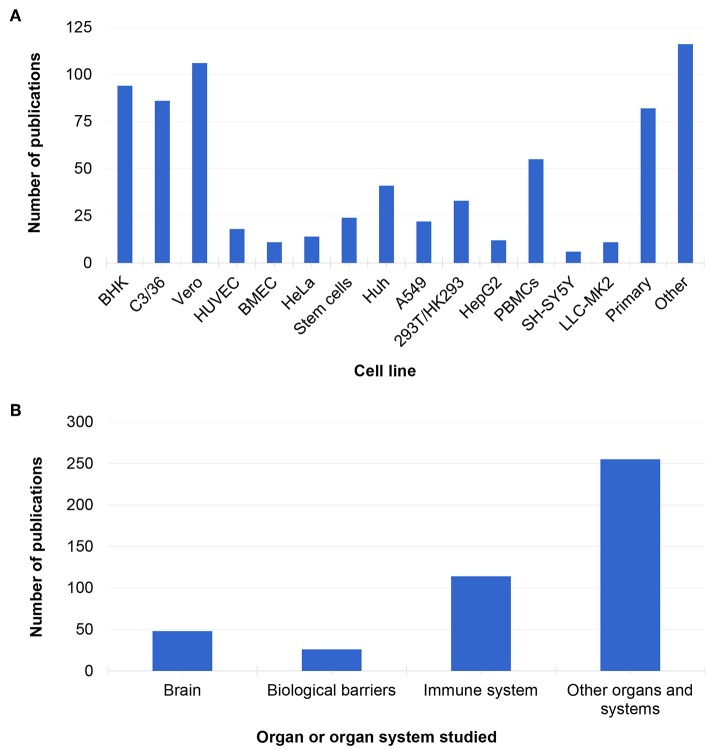
Cell lines **(A)** and organs and systems **(B)** represented in the *in vitro* literature.

Several cell lines have been used to study MBFs (Figure 2A). The most common cell lines used to study MBFs *in vitro* were African green monkey kidney epithelial cells (Vero cells, 106/412 studies), baby hamster kidney cells (BHK, 94/412 studies), and mosquito Aedes albopictus clone C6/36 cells (86/412 studies) (Figure 2A). This is because these three lines are often used to replicate viruses but not always to study pathogenesis, and often after replication, other cell systems or animal models are used (Azeredo et al., 2010; Hasebe et al., 2010; de Borba et al., 2012; Gandini et al., 2013).

The other cell lines which were prominent in the *in vitro* literature were primary cultures (mainly from mouse and human tissue, 82/412 studies) and peripheral blood mononuclear cell (PBMCs, 55/412 studies) (Figure 2A). Human PBMCs are a convenient source of human primary cells, because they can be obtained from both healthy volunteers and infected patients of all ages. These cells can also be used to study innate aspects of infection as well as the adaptive response to viral infection (Gunther et al., 2011; Correa et al., 2015; Rattanamahaphoom et al., 2017). In addition, dendritic cells, macrophages, and T cells can be derived from PBMCs for *in vitro* studies of the human immune response and host-pathogen interactions (Libraty et al., 2001; Reis et al., 2007; Wu et al., 2011; Gandini et al., 2013). In the future, blood isolated cell cultures could be use for personalized medicine. For example, monocytes and plasmacytoid dendritic cells (pDC) isolated from cryopreserved PBMC have been used to study the activation of pDC after DENV infection and their potential antiviral role (Gandini et al., 2013). However, one of the problems with PBMCs is the high genetic variability in the subjects. For instance, interferon responses (which are important for MBF research) vary depending on the individual, genre, and even populations (Whitney et al., 2003).

Stem cell-based models comprised only about 6% of the studies retrieved in this review (24/412 studies, Figure 2A). However, the use of induced pluripotent stem cells (iPSCs) has increased in recent years, and it is expected that this will be reflected in MBF research soon. Many of the studies that used iPSC cells were developed to study ZIKV in 3D *in vitro* models of the brain, such as brain organoids, spheroids, or other organotypic cultures (Dang et al., 2016; Gabriel et al., 2017). Such models have emerged in response to new epidemics of ZIKV in the Americas and around the world as well as in response to the need for novel tools to study virus pathogenesis and identify potential drug candidates with more human-relevant systems to study MBF infection.

*In vitro* literature was also categorized by organ or organ system studied (Figure 2B). Many studies focused on modeling the immune system, biological barriers such as the blood-brain barrier (BBB), and the brain (Figure 2B). Most of the *in vitro* studies utilized monolayer cultures (400/412 studies) and were performed on WNV and DENV, while only a few papers used 3D cultures (24/412 studies), and these were mostly focused on ZIKV (Cugola et al., 2016; Dang et al., 2016; Wells et al., 2016; Gabriel et al., 2017; Garcez et al., 2017; Zhou et al., 2017).

### Models of Brain Pathology in MBF Infection

ZIKV, DENV, and WNV infection can lead to severe neurological problems. These include aseptic meningitis, febrile convulsions in children, encephalitis, and myelitis (Turtle et al., 2012). Thus, models of the brain to study neuropathogenesis of MBF infection are needed. However, the brain is the most complex organ in the human body, and it is difficult to adequately model human neurophysiology. MBF neuropathogenesis has mainly been studied in monolayer cultures of various brain cell-type cancer cell lines, such as neuro-2a (de Borba et al., 2012; Ho et al., 2017), neuronal N18 (Su et al., 2001; Simanjuntak et al., 2015), primary cultures (Lucas et al., 2003; Diniz et al., 2006; Yoon et al., 2017; Zhu et al., 2017), and stem cell derived CNS cells (Shrestha et al., 2003; Retallack et al., 2016; Yoon et al., 2017). For example, a monolayer culture of primary microglia, astrocytes, and neurons isolated from a fetal human brain was used to understand the replicative properties of WNV in each cell population. It was determined that WNV mainly replicates in neurons and astrocytes and that following infection, both astrocytes and microglia were activated and produced cytokines and chemokines (Cheeran et al., 2005).

**Figure 3:**
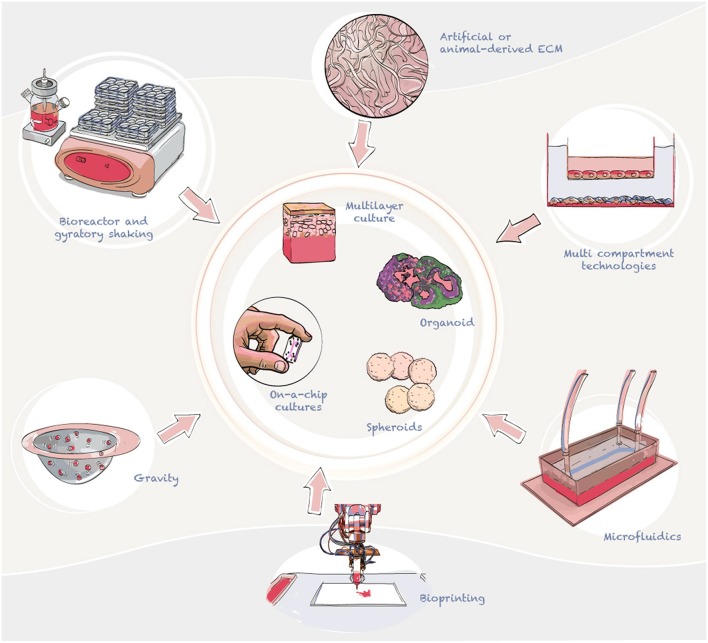
Advanced cell culture technologies currently employed to develop *in vitro* models of the brain and blood-brain barrier. In the diagram we summarize the main ways to generate 3D cultures, aggregating and multilayer technologies. Aggregation can be used to generate 3D cultures, for this gravity or shaking are used to form cell structures (normally spheric). Cells can also be plated to produce layers of different cell types to model tissues such as skin.

Over the last few years, we have seen an increase in the use of organotypic cell cultures (Pamies and Hartung, 2017), and specific models of the brain (Lancaster et al., 2013; Quadrato and Arlotta, 2017; Sloan et al., 2017). The recent outbreak of ZIKV has also generated an increase in the number of publications focused on brain models as a platform to study ZIKV (Cugola et al., 2016; Dang et al., 2016; Nowakowski et al., 2016; Gabriel et al., 2017; Garcez et al., 2017; Zhou et al., 2017). These models include neurospheres, brain organoids, 3D multilayer cultures of brain cell types, and brains-on-a-chip, among others. These 3D models can be generated through cell aggregation, by plating cells into an artificial or animal-derived extracellular matrix (ECM), such as a matrigel, or using a combination of methods. Cell aggregation can be accomplished with gravity (e.g., hanging drops and aggrewells) (Beauchamp et al., 2015), spinning using bioreactors (Qian et al., 2011), or gyratory shaking (Pamies et al., 2017) (Figure 3).

Advantages and disadvantages of 3D cultures have been summarized elsewhere (Alepee et al., 2014; Ravi et al., 2015; Fang and Eglen, 2017; Pamies and Hartung, 2017; Booij et al., 2019) including their use for ZIKV research (da Silva et al., 2018; Majolo et al., 2019). In summary, 3D models have shown to better reproduce the *in vivo* situation than 2D cultures. This is achived via the spatial conformation of 3D models allowing higher differentiation, survival and interaction between cells. In the case of brain models, 3D models allow growing neurons to interact with other cells for a longer period, which in 2D cultures is not possible, since these cells cultures tend to detach. Moreover, more complex models have been generated, but this complexity leads to other issues, such as lower reproducibility and higher researcher skill demands.

Neurospheres and organoids are the most popular 3D cultures used to study viral infection (18/24 studies on 3D models). While neurospheres consist of one cell type, such as neural precursors, neurons, or other CNS cells (Garcez et al., 2017), organoids present multiple cell types, and represent some aspect of organ structure and function (Dang et al., 2016; Nowakowski et al., 2016; Wells et al., 2016; Gabriel et al., 2017; Sacramento et al., 2017; Zhou et al., 2017).

The use of 3D models in MBF research has facilitated the study of mechanisms of MBF infection. For example, research using neurospheres of neural progenitor cells (NPCs) and cerebral organoids has demonstrated that NPCs and radial glia are more susceptible to altered cell cycle and cell death following ZIKV infection (Cugola et al., 2016; Dang et al., 2016; Nowakowski et al., 2016; Xu et al., 2016; Gabriel et al., 2017; Garcez et al., 2017; Zhou et al., 2017). Moreover, studies in cerebral organoids have shown that exposure to ZIKV at early stages of differentiation disrupts organoid expansion and fold formation (Li et al., 2017). Other cerebral organoid studies have shown that ZIKV targets and replicates in proliferating ventricular zone (VZ) apical progenitors, affecting NPC differentiation and leading to progenitor depletion, disruption of the VZ, impaired neurogenesis, and cortical thinning (Cugola et al., 2016; Gabriel et al., 2017). Organoids and neurospheres have also been used in drug development (Xu et al., 2016) and to explore the oncolytic potential of ZIKV (Zhu et al., 2017). Other iPSC-derived 3D models have also incorporated primary microglia cells to study flavivirus-microglia interaction in a multicellular system (Abreu et al., 2018). More complex models have the potential to better model human physiology than classical 2D *in vitro* models. In other fields, organoids have shown to be a novel tool for drug screening development for cancer (Weeber et al., 2017), intestinal diseases (Liu et al., 2016), and others.

There are also new models of the human brain that are being developed that have not yet been applied to MBF research but have potential applications in the field, such as brain region-specific organoids characterized by multiple components that correspond to the structures of various brain regions, such as the cerebral cortex (Kadoshima et al., 2014; Mariani et al., 2015; Pasca et al., 2015; Qian et al., 2016), midbrain (Jo et al., 2016; Qian et al., 2016), and hypothalamus (Merkle et al., 2015; Sakaguchi et al., 2015; Wang L. et al., 2015; Qian et al., 2016) (Figure 3). For example, cerebral organoids (also called mini brains) were initially described by Knoblich's laboratory (Lancaster et al., 2013). This model presented intrinsic self-organizing processes which display a cellular organization similar to early stages of the developing human brain. These feature allow scientists to study important key events during early brain development. The new tridimensional structure allows higher cell to cell interaction and maturation of the model. For example, Pasca's group have reported a method for generating pyramidal neurons from hiPSCs in a 3D cerebral cortex–like structure (Pasca et al., 2015). However, due to the lack of vasculature, necrotic centers form with in these tissues when the spheroid size is larger than 500 μm. Therefore, other models which are smaller in size but less complexity, have also been developed. For example. BrainSpheres developed in Hartung's laboratory have been able to recapitulate some of the key processes during CNS differentiation (Pamies et al., 2017). This model does not present spatial self-organization, however, has shown to be highly reproducible and presents a very high level of glial maturity in comparison with other models; showing maturate oligodendrocytes and partial axonal myelination (Pamies et al., 2017). There have also been different attempts to incorporate vascularization into the system to avoid hypoxia in the center of organoids (McGuigan and Sefton, 2006; Pham et al., 2018; Grebenyuk and Ranga, 2019; Homan et al., 2019). However, to date, vasculature formation has not been accomplished in 3D *in vitro* brain models.

In addition, less complex 3D organotypic cultures, such as multicellular spheres characterized by multiple cell types and more homogeneous structures that do not represent different brain regions (Von Tobel et al., 2016; Pamies et al., 2017), also have potential utility in MBF drug screening (Figure 3).

### Models of Biological Barriers

Brain endothelial cells are the most important barrier to prevent viral invasion and spread from the blood to the cerebral parenchyma. MBF infection frequently produces encephalitis, which requires crossing this blood-brain barrier (BBB). Two main types of *in vitro* BBB models have been developed: static (multilayer cultures, multicompartment cultures) and dynamic models (employing microfluidics) (Figure 3). Static models typically use 3D extracellular matrix platforms (Geltrex, Matrigel) and transwell plates, while dynamic models include a hollow fiber-based apparatus and/or microfluidic devices (Figure 3). Endothelial cell monolayers have been used widely with the purpose of studying pathophysiological changes that occur on the BBB. *In vitro* models of the BBB are not frequently used in the study of MBFs (seven studies on WNV, six studies on DENV, and only one study on ZIKV were retrieved in this literature review). Although the current volume of publications is low, BBB *in vitro* models are tremendously important to understanding MBF neuroinvasion. All the models identified in this literature review were static BBB models, but many other models that have recently been developed could be applied to MBF research (Brown et al., 2015; Cho et al., 2015; Wolff et al., 2015; van der Helm et al., 2016; Bang et al., 2017).

*In vitro* BBB models have been used to study membrane integrity following DENV infection of endothelial cells, and these studies demonstrated an increase in vascular permeability after DENV infection (Dewi et al., 2004; Talavera et al., 2004). In addition, DENV has been demonstrated to induce IL-6 and IL-8 production in human umbilical vein endothelial Cells (HUVECs), and these cytokines were found to produce apoptosis *in vitro* in the ECV304 human umbilical cord vein endothelial cell line (Avirutnan et al., 1998).

Endothelial cells have also been used to study WNV (Verma et al., 2009; Roe et al., 2014). The majority of papers retrieved involving BBB and WNV employed simplistic, monolayer cultures of human brain microvascular endothelial cells (HBMVEs). Although more complex systems are needed to better reproduce human physiology, these classical BBB models can help to confirm or hypothesize the virus' mechanism of infection (Verma et al., 2009, 2010) or pathogenesis (Durrant et al., 2014; Roe et al., 2014; Lazear et al., 2015).

ZIKV presented a limited bibliography, with only one paper for *in vitro* BBB models. In this study, HBMECs were grown in a rotating bioreactor to develop a human 3D cell-based model of the BBB microvascular endothelium characterized as a sphere of endothelial cells with a cavity inside (Bramley et al., 2017). Infection studies using this model demonstrated that ZIKV did not easily infect the HBMEC model, but that exposure to TNF-α could enhance infection (Bramley et al., 2017). ZIKV has also been studied using models of other biological barriers, such as the blood-testis barrier (Siemann et al., 2017), and the umbilical barrier (Jacobs and Levin, 2002; Raekiansyah et al., 2014).

### Models of the Immune System

Sometimes, pathogens can compromise the BBB and enter the brain producing an inflammatory response, which typically involves the activation of microglia, the resident mononuclear phagocytes of the brain (Ginhoux et al., 2013). Microglia are responsible for the production of cytokines and other molecules that regulate inflammation and other homeostatic responses in brain, and these conditions can lead to brain diseases (Dheen et al., 2007). There are also other cells in the CNS that interact with the immune system such oligodendrocytes, astrocytes, endothelial cells, perivascular macrophages and some types of neurons. These cells produce cytokines and chemokines that interact with the immune system under inflammatory response.

The immune system consists of complex interactions of a variety of cells, including monocytes, leukocytes, dendritic cells, platelets, and stem cells, among others. PBMCs are the most commonly used because human blood is a very convenient cell source that can be stored easily. Although PBMCs consist of a mixed population of blood leukocytes, they can also be used as a primary souce to futher purification of specific cell types (e.g., T cells, B cells) (Reis et al., 2007; Wu et al., 2011; Rawle et al., 2015; Zhang J. et al., 2016; Rattanamahaphoom et al., 2017).

Immune system models found in this literature review were mainly used to study DENV, with fewer studies found for WNV or ZIKV. Most *in vitro* studies on MBF infection and the immune system were focused on viral molecular mechanisms. However, a few papers were focused on neuroinflammation. For example, human macrophages were used to study the role of Sema7, a membrane-associated/secreted protein that plays an essential role in connecting the vertebrate neuronal and immune systems in WNV pathogenesis (Sultana et al., 2012). The majority of papers studying neuroinflammation are *ex vivo* or *in vivo* models. There is therefore an urgent need to generate better *in vitro* models that represent the complex relationships between the brain and immune system and that can more accurately model neuroimmune responses. The development of new *in vitro* brain models incorporating microglia have the potential to become an important tool to study neuroinflammation following MBF infection. For example, recently microglia have been incorporate into a 3D iPSC derived model in order to study inflammation after ZIKV infection (Abreu et al., 2018). However, integrating the wider, peripheral immune system with the brain remains one of the major challenges for *in vitro* technologies.

Overall, *in vitro* models have proved to be a useful tool to study MBF, with examples that study virus molecular mechanisms (Correa et al., 2015; Londono-Renteria et al., 2017; Rattanamahaphoom et al., 2017), their implication in the inflammatory response (Lim et al., 2014; Duran et al., 2016), drug screening (Stolp et al., 2015; Wang L. F. et al., 2015; Sayce et al., 2016), or vaccine development (Nantachit et al., 2016, 2017; Kam et al., 2017). Thus, human cell-derived *in vitro* models could present a solution to modeling MBF infection and neuropathogenesis.

## *In silico* Studies

Efficient clinical translation of basic research findings is of paramount importance to protecting public health with regard to outbreaks of MBF infections. However, the traditional approach to development and testing of new biomedical products using *in vitro* or *in vivo* animal studies in the early stages of pre-clinical drug development can be time consuming and prohibitively expensive. *In silico* methods allow researchers to interpret large amounts of biological data efficiently, reduce costs, and speed up the early stages of drug development. The use of *in silico* modeling tools in MBF research has already aided in the identification of disease transmission patterns, allowed researchers to predict risk factors associated with the spread of MBF infection, and molecular modeling has advanced the mechanistic understanding of host-pathogen interactions at the molecular level as well as allowed researchers to identify potential vaccine candidates and therapeutic strategies. Such studies represent promising advancements in the field of MBF research and indicate great potential for an efficient, inexpensive, comprehensive, and mechanistically-driven drug candidate identification process.

In this literature review, *in silico* modeling and other computational techniques were shown to be valuable to MBF research and a promising direction for the field moving forward. *In silico* studies retrieved in the literature review were categorized by applicability to MBF type (DENV, ZIKV, WNV) (Figure 1B). The majority of *in silico* studies were focused on DENV (180/253 studies), followed by WNV (75/253 studies), and ZIKV (66/253 studies) (Figure 1B). There were also studies labeled with more than one MBF type because many of the *in silico* models (39/253 studies) were found to have general applicability to all or many types of flaviviruses (e.g., studies based on models of a shared protein structure or transmission dynamics modeling of a vector host species which carries more than one type of MBF). The *in silico* literature was also sorted by study objective into the following categories: vector control, vaccine development, transmission dynamics, antiviral therapy development, mosquito-virus interactions, or human pathogenesis (Figure 1C). Most studies were labeled as either antiviral therapy development studies (99/253 studies) or transmission dynamics studies (91/253 studies), followed by vaccine development studies (24/253), vector control studies (23/253 studies), human pathogenesis research (22/253 studies), and mosquito-virus interaction studies (4/253 studies) (Figure 1C).

### Antiviral Therapy Development Studies

Antiviral agents for MBF infection have generally been designed to inhibit viral entry, replication, or the host factors that permit viral infection. Thus, much of the computational virology literature on antiflaviviral drug development has focused on molecular modeling of the structures that play a role in these processes, such as the viral envelope, capsid, and enzymes, as well as the interacting host factors, in order to determine the optimal targets for drug development, screen compounds for potential antiviral activity, and identify the most promising therapeutic candidates for further testing.

A number of studies focused on *in silico* screening of potential inhibitory compounds that bind with high affinity to either the non-structural protein 5 (NS5) methyltransferase (MTase) domain (Milani et al., 2009; Podvinec et al., 2010; Stephen et al., 2016) or the RNA dependent RNA polymerase domain of DENV (Anusuya et al., 2016; Elfiky, 2016; Manvar et al., 2016), both of which play key roles in viral replication within host cells. In addition, many *in silico* molecular docking studies have been instrumental in screening compounds that target either the NS3 helicase domain that unwinds the viral RNA for genome replication, or the protease domain that cleaves viral precursor proteins (Pambudi et al., 2013; Parida et al., 2013; Wichapong et al., 2013; Mukhametov et al., 2014; Viswanathan et al., 2014; Dwivedi et al., 2016; Shiryaev et al., 2017). There have also been studies which focus on screening a particular class of drug candidate for potential binding to and neutralizing of many key viral enzymes, such as the recent study in which phytochemicals were screened for their binding affinity to the structures of the ZIKV protease, helicase, methyltransferase, and polymerase (Byler et al., 2016).

The structural proteins of the viral envelope are also important targets to explore in antiflaviviral drug discovery as a strategy to inhibit viral entry into host cells. Many studies have developed molecular docking approaches to screen compounds for their ability to bind to the viral envelope proteins of DENV (Li et al., 2008; Zhou et al., 2008; Wang et al., 2009; Yennamalli et al., 2009). Computational approaches have been used to build on naturally occurring DENV sequences, predicting novel peptide structures that display increased target binding affinity and improved inhibition of viral binding and entry while maintaining low host cell toxicity (Costin et al., 2010). More recently, a homology model of the ZIKV envelope protein was resolved, and a simulation-based approach was used to identify the binding sites of potential small molecule inhibitors (Fernando et al., 2016). There are also host factors that play a role in viral infection and can be targeted in antiviral therapy. *In silico* approaches have been used for virtual screening of small molecule inhibitors of the human helicase X-linked DEAD-box polypeptide 3 (DDX3), a protein which interacts with viral proteins and allows viral replication to occur within host cells (Fazi et al., 2015).

*In silico* methods have also been used following *in vivo* or *in vitro* testing to elucidate the mode of action or binding site of particular antiviral candidates. For example, structure modeling was used to demonstrate that a monoclonal antibody that neutralizes DENV and WNV *in vitro* and in *in vivo* mouse studies binds to the fusion loop peptide of the flavivirus envelope protein (Deng et al., 2011) and to confirm *in vivo* and *in vitro* evidence that an antiviral compound binds to and inhibits the DENV capsid protein (Scaturro et al., 2014). Structure-activity relationships have also been used to identify the inhibitory functional groups of a DENV NS2B-NS3 protease inhibitor identified with cell culture experiments (Tomlinson and Watowich, 2011) and molecular modeling of antimalarial compounds was used to understand the stereo-electronic properties that allowed these compounds to also confer DENV antiviral activity in cell culture (Balasubramanian et al., 2017).

A number of *in silico* studies on antiflavivirals have been used to inform further *in vitro* and *in vivo* testing. For example, molecular modeling of the protease-inhibitor complex of potential ZIKV antivirals was used to identify structural scaffolds for allosteric small-molecule inhibitors of the ZIKV NS2B-NS3 protease, and the most potent inhibitors were then tested both *in vitro* in human fetal neural progenitor cells and *in vivo* in mice and found to demonstrate inhibition of ZIKV propagation (Shiryaev et al., 2017).

Overall, the recent computational studies on antiviral drug candidates demonstrate the potential of *in silico* tools to aid in the rational design of antiflavivirals, identify pharmaceutically relevant drug targets, and accelerate the process of antiflaviviral drug development. A good example of such trend is the OpenZika project, in which components of a software system are shared among multiple sources (i.e., distribures computing) to screen potential anti-ZIKV compounds with increased efficiency, using publicly accessible data (Ekins et al., 2016). It demonstrates that there are many future opportunities to increase the scale, rate, and transparency with which new antivirals are identified in the future with *in silico* tools. As timing is crucial in the development of therapies for emerging diseases, *in silico* models that facilitate the prioritization of drug candidates prior to testing are particularly relevant to emerging flaviviral infections and establish *in silico* methods as a valued resource in the development of antiviral therapies.

### Vaccine Development Studies

Vaccine development is a relatively new application of *in silico* methods within MBF research. Recently, computational tools and immunoinformatics approaches have been developed for virtual screening of antigen sequences to predict epitopes that can act as vaccine targets. *In silico* docking studies and molecular dynamics simulations have been conducted as a first step in the design of epitope-based peptide vaccines for DENV (Lund et al., 2011; Jones et al., 2017), WNV (Larsen et al., 2010), and ZIKV (Alam et al., 2016; Dikhit et al., 2016; Ali et al., 2017). In addition, bioinformatics software and neural networks were used in a recent study to evaluate a tetravalent protein subunit vaccine for all four serotypes of DENV (Fahimi et al., 2016). A mathematical model that simulates many of the cellular and protein dynamics involved in the host immune response was also recently developed as a tool to model vaccination effects (Bonin et al., 2017) and could become a useful strategy for vaccine development in combination with other pre-screening methods.

Immunoinformatics and *in silico* screening approaches such as these have potential to reduce the amount of time and resources needed for epitope identification compared to traditional peptide screening methods, and have demonstrated their usefulness in the initial stages of vaccine design, such as in pre-screening of potential vaccine candidates. Such prediction models could allow vaccine candidates with greater immunological relevance against emerging infections to be identified at a rapid rate in order to speed up the process of vaccine development for emerging MBF infections.

### Human Pathogenesis Research

Although the majority of molecular mechanisms responsible for the neurotropism of MBF infection remain unknown, *in silico* methods are emerging as promising tools to aid in the investigation of host-pathogen interactions that contribute to pathogenesis of MBF infection. An *in silico* genetic screening approach was recently utilized to elucidate that ZIKV-elicited congenital microcephaly may act through dysregulation of the retinoic acid response element (RARE) (Kumar et al., 2016). In addition, *in silico* analysis has been employed to investigate the role of both viral and human microRNA in congenital ZIKV microcephaly (Pylro et al., 2016).

In addition to studies focused on ZIKV-associated microcephaly, *in silico* tools have also been used to explore the mechanism by which DENV infects and replicates within host cells (Anglero-Rodriguez et al., 2014), and to predict human protein interactions with DENV (Doolittle and Gomez, 2011). Computational methods have also been used to identify patterns in inter-individual variation in DENV viral load following infection (Ben-Shachar et al., 2016). Also, *in silico* models can use protein sequence information to predict intrinsic disorder within the viral proteome, indicating where proteins lack a fixed 3D structure and display functional diversity and structural plasticity. Computer-based prediction of the intrinsically disordered proteins (IDP) in DENV revealed a positive correlation between the level of IDP in DENV and WNV capsid and membrane proteins and virulence (Goh et al., 2016). Observations that the most disordered proteins are associated with the most virulent viruses are crucial for designing novel drug targets and in enabling a greater understanding of virus attenuation needed for vaccine development.

### Transmission Dynamics and Vector Control Studies

Much of *in silico* MBF research focuses on modeling transmission and outbreak dynamics to predict epidemics and model prevention and mitigation strategies. In this review, over one-third of the *in silico* studies were labeled as transmission dynamics studies. Many studies have utilized climate- or meteorological-based simulations of transmission by birds and mosquitoes along with other epidemiological and vector life cycle model inputs to predict transmission of WNV (Bouden et al., 2008; Laperriere et al., 2011; Manore et al., 2014) and DENV (Fan et al., 2010; Chowell et al., 2011; Otero et al., 2011; Campbell et al., 2015; Morin et al., 2015; Chanprasopchai et al., 2017;Siraj et al., 2017).

*In silico* transmission dynamics studies have incorporated non-traditional data sources into epidemic prediction models, such as Google search trends or other web-based data sources for epidemics of DENV (Yang et al., 2017) and ZIKV (Majumder et al., 2016; Wang et al., 2016; McGough et al., 2017; Teng et al., 2017). *In silico* studies such as these allow for an understanding of the timing and magnitude of MBF epidemics, as well as the environmental factors which can influence transmission. Many studies also incorporated potential vaccination strategies for DENV, to evaluate potential effectiveness of various vaccine scenarios given transmission dynamics typical to an endemic region and justify vaccination programs (Thavara et al., 2014; Knipl and Moghadas, 2015; Hladish et al., 2016).

*In silico* tools were also used to screen potential larvicides and insecticides to control vector species (Dhananjeyan et al., 2009; Oliferenko et al., 2013; da Silva et al., 2015; Canizares-Carmenate et al., 2017; Silva et al., 2017) and to model vector dynamics or transmission patterns considering the effect of vector genetic manipulation strategies (Atkinson et al., 2007; Huang et al., 2007; Rasgon, 2009; Okamoto et al., 2013), vector infection with Wolbachia, an endosymbiotic bacteria that prevents viral transmission (Zhang X. et al., 2016; Xue et al., 2017), vector control chemicals, such as larvicides and insecticides (Hu et al., 2009; Oki et al., 2011; Pawelek et al., 2014) or the use of vector control chemicals along with other strategies, such as the destruction of mosquito breeding sites (Amaku et al., 2014).

### Mosquito-Virus Interaction Studies

The study of mosquito-virus interactions, which was represented in very few *in silico* studies that were reviewed (Figure 1C), is fundamental for MBF research because these viruses must replicate in mosquito cells in order to continue their transmission cycle. The studies in this category focused on assembling genomic and gene expression databases or computational analysis tools that allow the study of interactions between the viral and vector genome (Dissanayake et al., 2010; Dudchenko et al., 2017). Others focused on the protein-protein interactions that occur when an MBF infects one of its mosquito host species (Guo et al., 2010; Doolittle and Gomez, 2011). Leveraging computational databases and tools such as these can aid in the development of novel vector control strategies, such as genetic modification.

## Discussion

MBF infection is considered a major public health concern, and DENV, ZIKV and WNV, in particular, present a high burden of associated neurological disease. Neuropathogenesis associated with MBF infection is often human-specific, and thus, it has been difficult to find adequate models to study treatments and prevention strategies. Understanding the advantages and limitations of models used in MBF reserch is important in obtaining good quality data (Table 2). There is a need for additional scientific interest and funding to increase the rate of post-mortem studies that emphasize pathophysiology. There is also a lack of prospective studies that follow individuals infected with MBFs. Such studies could provide insight into the progression of signs and symptoms alongside molecular and serology studies. Moreover, studies on naturally infected patients could aid in the investigation of biological samples other than blood that could be used to diagnose MBF infection and elucidate MBF reservoirs (e.g., urine in ZIKV infection).

**Table 2:** Problems and possible solutions for MBF research.

**Study type**	**Problems**	**Solutions**
Human	Difficult to study progression of disease	Development of multidisciplinary networks (health care workers, virologists, epidemiologists) that work together with local registries to improve disease surveillance following flavivirus infection.
	Restricted access to pathological studies	Development of biobanks of samples obtained from sick patients in different stages of disease. Increased prospective controlled studies in naturally infected population.
*In vivo*	Human relevance	Use or combine *in vivo* studies with *in silico* or human *in vitro* models.
	Cost	Combination with computational and *in vitro* models
*In vitro*	Reproducibility	Increased rigor and application of GCCP Standardization of *in vitro* methods
	Human relevance	Use of more complex systems (organoids, microfluidic organ-on-chips, etc.) Use of primary human cells
*In silico*	Often needs validation using other methods (*in vitro* or *in vivo*)	Produce better predictive models Increase confidence in models

Although NHPs have been used extensively to study MBFs, many of the potential vaccine and antiviral drugs that were successful in NHP preclinical research have failed in human clinical trials (see Supplementary Table 1). Other animal models, such as mice, have also been used as a low cost alternative to NHPs, and procedures exist to humanize mice to increase their susceptibility to infection. However, both mouse models and NHPs are still limited in their physiological relevance, and thus, results from these studies may not translate to human clinical studies. *In vitro* and *in silico* models have potential to overcome the limitations and ethical concerns associated with *in vivo* animal studies, but these methods also present unique challenges. *In vitro* models have been used in many MBF studies to elucidate molecular mechanisms underlying infection and pathogenesis. However, *in vitro* models that rely on cancer cell lines or monolayer cultures are limited in human relevance. Stem cell-derived models should be considered optimal human cell sources, together with primary human cell cultures and peripheral blood samples when possible, and GCCP should always be practiced (Coecke et al., 2005; Pamies and Hartung, 2017). In addition, novel cell culture systems, such as spheroids, organoids, microfluidic systems, and bioprinted multi-layered cultures that allow the study of cell-cell and cell-matrix interactions in a 3D *in vitro* environment have demonstrated potential to increase the physiological relevance of *in vitro* studies.

Overall, *in silico* methods have proven to be valuable resources in MBF research, to design and screen large libraries of potential antiviral and vaccine candidates as well as model host-pathogen interactions and study potential mechanisms of infection and transmission dynamics. *In silico* models can play a major role in research on the prevention of MBF infection through vector control and provide a better understanding of MBF transmission factors and patterns that lead to widespread MBF outbreaks. In addition, *in silico* modeling and high throughput screening of therapeutic or preventative drug candidates followed by *in vitro* validation has potential to speed up the process of MBF drug development.

Thus, far, both *in silico* and *in vitro* methods have primarily been developed to generate preliminary data that would typically be followed by *in vivo* testing to validate findings. This significantly reduces the amount of animal testing required for MBF research but does not completely replace animal testing. In the long-term, it would be advantageous to further develop *in silico* and *in vitro* methods with the goal of replacement in mind, because often, preclinical success in animal models does not translate to efficacy in human clinical trials, and failure in an animal model does not necessarily indicate a lack of human efficacy. Logically, the failure of certain drugs or vaccines when moving from *in vitro* to pre-clinical *in vivo* or from pre-clinical animal models to humans is often a function of specific pharmacological properties of potential drugs, and replacing animal models will require additional novel *in vitro* or *in silico* approaches that can accurately predict bioavailability and stability of drug candidates. However, a combination of *in vitro* and *in silico* methods have the potential to bridge this translational gap between preclinical and clinical research, and more integrated approaches combining or linking different studies and methods are essential to better understand the mechanism of MBF infection and resulting diseases and improve the drug development process.

We envision a future of MBF research with less reliance on NHP and rodent models and an increase in the number and rigor of *in vitro* and *in silico* studies. The emergence of new technologies (e.g., induced pluripotent stem cell models, organotypic cultures, and high throughput *in silico* screening) will help to better predict human pathogenesis and drug efficacy. We believe that harmonization of methods and centralization of data, together with combining different approaches to reach a common objective, are crucial to better understand MBF infection and neuropathogenesis and obtain clinical solutions.

## Author Contributions

The literature review and classification of the papers were done by MC, LM, GH, DF, and DP. Most of the writing and figures have been performed by MC, DP, and LM. LG and CP have revised and advice in the structure of the document due to the extensive knowledge in the topic.

### Conflict of Interest Statement

The authors declare that the research was conducted in the absence of any commercial or financial relationships that could be construed as a potential conflict of interest.
