# Supplementary material for: In vitro and in silico Models to Study Mosquito-Borne Flavivirus Neuropathogenesis, Prevention, and Treatment
Source: Front Cell Infect Microbiol. 2019 Jul 9;9:223. doi: 10.3389/fcimb.2019.00223 (PMC6629778; doi:10.3389/fcimb.2019.00223)
Supplement: Supplementary file 1 [file Table_1.DOCX]

**Table 1. Clinical trials.**

| Author | Phase | Vaccine | Location | Population | Efficacy | Adverse effects |
| --- | --- | --- | --- | --- | --- | --- |
| (Capeding et al., 2014) | I | CYD-TDV | 5 countries in Asia-Pacific | Healthy children 2-14 years | Reduced cases in 56.5% | Infections and injuries |
| (Watanaveeradej et al., 2014) | II | live-attenuated tetravalent dengue virus (TDEN) vaccine | Thailand | 120 healthy, predominantly flavivirus-primed adults | All flavivirus-unprimed subjects and at least 97.1% of flavivirus-primed subjects were seropositive to antibodies against all four DENV types 1 and 3 months post-TDEN dose 2. | There were no vaccine-related serious adverse events or dengue cases reported |
| (Villar et al., 2013) | II | recombinant, live-attenuated, tetravalent dengue vaccine (CYD-TDV) | Latin America | 9-16 year olds | After the third dose of CYD-TDV, 100%, 98.6% and 93.4% of participants were seropositive for at least 2, at least 3 or all 4 serotypes, respectively. | Safety |
| (Durbin et al., 2013) |  | 4 different admixtures of a live attenuated tetravalent (LATV) dengue vaccine |  | 113 flavivirus-naive adults | induced a trivalent or better neutralizing antibody response in 75%-90% of vaccinees.  TV003 induced a trivalent or greater antibody response in 90% of flavivirus-naive vaccinees and is a promising candidate for the prevention of dengue. | no significant difference in the incidence of adverse events between vaccinees and placebo-recipients other than rash. |
| (Sabchareon et al., 2012) | IIb | CYD-TDV | Thai | healthy Thai schoolchildren aged 4-11 years | Efficacy was 30·2% (95% CI -13·4 to 56·6), and differed by serotype. | Dengue vaccine was well tolerated, with no safety signals after 2 years of follow-up after the first dose |
| (Leo et al., 2012) | II | CYD-TDV | Singapure |  | Reactogenicity after subsequent CYD-TDV doses was no higher than after the first dose, and tended to be lower or similar to that seen after active control vaccination.  Post-dose 3, 66.5% of all participants were seropositive to all four serotypes, and 87.2% were seropositive to ≥ 3 serotypes | Safe |
| (Capeding et al., 2011) | I | CYD-TDV | Philippines | groups of participants aged 2-5, 6-11, 12-17, and 18-45 years | After three TDV vaccinations, the seropositivity rates against serotypes 1-4 were: 91%, 100%, 96%, 100%, respectively, in 2-5 year-olds; 88%, 96% 96%, 92% in 6-11 year-olds; 88%, 83%, 92%, 96% in adolescents; and 100% for all serotypes in adults.  The safety profile of TDV in a flavivirus endemic population was consistent with previous reports from flavivirus naïve populations. | No serious adverse vaccine related events and no significant trends in biological safety parameters were reported. |
| (Beckett et al., 2011) | I | dengue virus serotype-1 (DENV-1) vaccine construct (D1ME(100))  DNA vaccine | United States | 22 healthy flavivirus-naïve adults assigned to one of two groups. | Five subjects (41.6%) in the high dose group and none in the low dose group developed detectable anti-dengue neutralizing antibodies. | The safety profile of the DENV-1 DNA vaccine is acceptable at both doses administered in the study. |
| (Poo et al., 2011) | I | CYD-TDV | Mexico City | children aged 2 to 5, 6 to 11, and 12 to 17 years (36 children per age group), and adults (n = 18) aged <45 years – DENV naïve | Reactogenicity did not increase with successive TDV injections  After 3 TDV vaccinations, the seropositivity rate against each dengue serotype was in the range 77% to 92%, compared with 85% to 94% after completion of the YF-TDV-TDV regimen. Of the 2- to 11-year-old participants, 95% were seropositive against ≥3 serotypes after 3 vaccinations. | No vaccine-related serious adverse events, |
| (Wright et al., 2009) | I | rDEN4Delta30-4995 is a live attenuated dengue virus type 4 (DENV4) vaccine | Vanderbilt University Medical Center | 28 healthy adult volunteers | None of the rDEN4Delta30-4995 vaccinees became viremic, yet 95% developed a four-fold or greater increase in neutralizing antibody titers. | The vaccine was safe, well-tolerated, and immunogenic. An asymptomatic generalized maculopapular rash and elevations in ALT levels were observed in 10% of the rDEN4Delta30-4995 vaccinees. |
| (Sun et al., 2009) | II | TDV – 3 formulations |  | 71 healthy adult subjects | Thirty-six percent, 40% and 63% of vaccinated subjects developed tetravalent neutralizing antibodies after two doses of Formulations 13, 14 and 17, respectively. Formulation 17 was selected for further clinical evaluation based on this study. | Formulation 13 was the most reactogenic, while both Formulations 14 and 17 were similar in reported reactions. |
| (Guirakhoo et al., 2006) | I | yellow fever (YF)-dengue 2 (DEN2) chimera (ChimeriVax-DEN2) | US | Forty-two healthy YF naïve adults randomly received a single dose of either ChimeriVax-DEN2 (high dose, 5 log plaque forming units [PFU] or low dose, 3 log PFU) or YF-VAX by the subcutaneous route | These data demonstrated that (1) the safety and immunogenicity profile of the ChimeriVax-DEN2 vaccine is consistent with that of YF-VAX, and (2) preimmunity to YF virus does not interfere with ChimeriVax-DEN2 immunization, but induces a long lasting and cross neutralizing antibody response to all 4 DEN serotypes. | Most adverse events were similar to YF-VAX and of mild to moderate intensity, with no serious side-effects. |
| (Edelman et al., 2003) | I | Tetravalent vaccines  in 16 dosage combinations |  | 64 flavivirus non-immune adult volunteers | Similar proportions of volunteers seroconverted to dengue-1 (69%), dengue-2 (78%), and dengue-3 (69%), but significantly fewer volunteers seroconverted to dengue-4 (38%). The geometric mean 50% plaque reduction neutralization test titers in persons who seroconverted were significantly higher to dengue-1 (1:94) than to dengue-2 (1:15), dengue-3 (1:10), and dengue-4 (1:2). |  |

Beckett, C.G., Tjaden, J., Burgess, T., Danko, J.R., Tamminga, C., Simmons, M., et al. (2011). Evaluation of a prototype dengue-1 DNA vaccine in a Phase 1 clinical trial. *Vaccine* 29(5)**,** 960-968. doi: 10.1016/j.vaccine.2010.11.050.

Capeding, M.R., Tran, N.H., Hadinegoro, S.R., Ismail, H.I., Chotpitayasunondh, T., Chua, M.N., et al. (2014). Clinical efficacy and safety of a novel tetravalent dengue vaccine in healthy children in Asia: a phase 3, randomised, observer-masked, placebo-controlled trial. *Lancet* 384(9951)**,** 1358-1365. doi: 10.1016/S0140-6736(14)61060-6.

Capeding, R.Z., Luna, I.A., Bomasang, E., Lupisan, S., Lang, J., Forrat, R., et al. (2011). Live-attenuated, tetravalent dengue vaccine in children, adolescents and adults in a dengue endemic country: randomized controlled phase I trial in the Philippines. *Vaccine* 29(22)**,** 3863-3872. doi: 10.1016/j.vaccine.2011.03.057.

Durbin, A.P., Kirkpatrick, B.D., Pierce, K.K., Elwood, D., Larsson, C.J., Lindow, J.C., et al. (2013). A single dose of any of four different live attenuated tetravalent dengue vaccines is safe and immunogenic in flavivirus-naive adults: a randomized, double-blind clinical trial. *J Infect Dis* 207(6)**,** 957-965. doi: 10.1093/infdis/jis936.

Edelman, R., Wasserman, S.S., Bodison, S.A., Putnak, R.J., Eckels, K.H., Tang, D., et al. (2003). Phase I trial of 16 formulations of a tetravalent live-attenuated dengue vaccine. *Am J Trop Med Hyg* 69(6 Suppl)**,** 48-60.

Guirakhoo, F., Kitchener, S., Morrison, D., Forrat, R., McCarthy, K., Nichols, R., et al. (2006). Live attenuated chimeric yellow fever dengue type 2 (ChimeriVax-DEN2) vaccine: Phase I clinical trial for safety and immunogenicity: effect of yellow fever pre-immunity in induction of cross neutralizing antibody responses to all 4 dengue serotypes. *Hum Vaccin* 2(2)**,** 60-67.

Leo, Y.S., Wilder-Smith, A., Archuleta, S., Shek, L.P., Chong, C.Y., Leong, H.N., et al. (2012). Immunogenicity and safety of recombinant tetravalent dengue vaccine (CYD-TDV) in individuals aged 2-45 y: Phase II randomized controlled trial in Singapore. *Hum Vaccin Immunother* 8(9)**,** 1259-1271. doi: 10.4161/hv.21224.

Poo, J., Galan, F., Forrat, R., Zambrano, B., Lang, J., and Dayan, G. (2011). Live-attenuated Tetravalent Dengue Vaccine in Dengue-naive Children, Adolescents, and Adults in Mexico City: Randomized Controlled Phase 1 Trial of Safety and Immunogenicity. *Pediatr Infect Dis J* 30(1)**,** e9-17. doi: 10.1097/INF.0b013e3181fe05af.

Sabchareon, A., Wallace, D., Sirivichayakul, C., Limkittikul, K., Chanthavanich, P., Suvannadabba, S., et al. (2012). Protective efficacy of the recombinant, live-attenuated, CYD tetravalent dengue vaccine in Thai schoolchildren: a randomised, controlled phase 2b trial. *Lancet* 380(9853)**,** 1559-1567. doi: 10.1016/S0140-6736(12)61428-7.

Sun, W., Cunningham, D., Wasserman, S.S., Perry, J., Putnak, J.R., Eckels, K.H., et al. (2009). Phase 2 clinical trial of three formulations of tetravalent live-attenuated dengue vaccine in flavivirus-naive adults. *Hum Vaccin* 5(1)**,** 33-40.

Villar, L.A., Rivera-Medina, D.M., Arredondo-Garcia, J.L., Boaz, M., Starr-Spires, L., Thakur, M., et al. (2013). Safety and immunogenicity of a recombinant tetravalent dengue vaccine in 9-16 year olds: a randomized, controlled, phase II trial in Latin America. *Pediatr Infect Dis J* 32(10)**,** 1102-1109. doi: 10.1097/INF.0b013e31829b8022.

Watanaveeradej, V., Gibbons, R.V., Simasathien, S., Nisalak, A., Jarman, R.G., Kerdpanich, A., et al. (2014). Safety and immunogenicity of a rederived, live-attenuated dengue virus vaccine in healthy adults living in Thailand: a randomized trial. *Am J Trop Med Hyg* 91(1)**,** 119-128. doi: 10.4269/ajtmh.13-0452.

Wright, P.F., Durbin, A.P., Whitehead, S.S., Ikizler, M.R., Henderson, S., Blaney, J.E., et al. (2009). Phase 1 trial of the dengue virus type 4 vaccine candidate rDEN4{Delta}30-4995 in healthy adult volunteers. *Am J Trop Med Hyg* 81(5)**,** 834-841. doi: 10.4269/ajtmh.2009.09-0131.
