# Supplementary material for: In vitro and in silico Models to Study Mosquito-Borne Flavivirus Neuropathogenesis, Prevention, and Treatment
Source: Front Cell Infect Microbiol. 2019 Jul 9;9:223. doi: 10.3389/fcimb.2019.00223 (PMC6629778; doi:10.3389/fcimb.2019.00223)
Supplement: Supplementary file 2 [file Table_2.DOCX]

**Table 2. Animal models: neuropathogenesis**

| Author | Virus type | Model | Findings |
| --- | --- | --- | --- |
| (Shao et al., 2017) | DENV-2 and ZIKV | Embryonic mouse brains (intracerebral inoculation) | DENV-2 infection caused smaller brain size due to neural progenitor cells´ death. ZIKV was more potent in causing brain damage and postnatal lethality. |
| (Kuszpit et al., 2017) | ZIKV | Immunocompromised C57BL/6 mice (intraperitoneal inoculation) | ZIKV infection caused global brain inflammation detected by PET scan imaging. Pathology revealed widespread encephalitis (neuronal degeneration and necrosis) with minimal microgliosis; minimal perivascular edema and hemorrhage. |
| (Yoon et al., 2017) | ZIKV | Embryonic mouse brains (intracerebral inoculation) | ZIKV-NS2A, but not DENV-NS2A, reduced proliferation and premature differentiation of radial glial cells and aberrant positioning of newborn neurons. |
| (Duggal et al., 2017) | ZIKV | Immunocompetent CD-1/ICR adult mice (intracerebral inoculation) | ZIKV strain from Uganda exhibited 80-100% mortality rate; ZIKV strain from Senegal and Puerto Rico caused 30% and no mortality, respectively. |
| (Bortell et al., 2017) | WNV | C57BI/6 and C57BI/6 Osteopontin knock out (intraperitoneal inoculation) | Osteopontin expression controls apoptosis of WNV infected cells in the brain and prevents lethal viral spread. |
| (Wang et al., 2017) | ZIKV | C57BL/6 (Axl-/- and Axl+/-) (intracerebral inoculation) | Axl, a receptor tyrosine kinase, is not an indispensable factor for ZIKV infection in the brain and other organs. |
| (Ho et al., 2017) | DENV-2 (PL046) | 7-day ICR (immunocompetent) mice; intracerebral and intraperitoneal inoculation | DENV infected neurons (Neuro-2a cells) in vivo, and caused neurotoxicity, cell grow inhibition and apoptosis (7 days post-infection). Neurological findings were present in infected mice and absent in mock. Inhibition of endosomal acidification increased the survival rate in infected mice. |
| (Ho et al., 2017) | DENV-2 (PL046) | 7-day ICR (immunocompetent) mice; intracerebral and intraperitoneal inoculation | Treatment with PCZ, a D2R antagonist, and Pitstop, a clathrin inhibitor, decreased neurotoxicity (in-vitro). The use of BafA1 and ConA, inhibitors of V-ATPase to decease endosomal acidification, blocked DENV-2 dsRNA expression. |
| (Oh et al., 2017) | ZIKV | A129 (IFNr-/-) (intraperitoneal and intracranial inoculation) | ZIKV infected dorsal root ganglia, gut and spinal cord neurons. |
| (Paul et al., 2017) | WNV | WT mice (Osteopontin-/-) (intraperitoneal inoculation) | Osteopontin facilitated WNV neuroinvasion by recruiting WNV-infected polymorphonuclear into the mouse brain. |
| (Yu et al., 2017) | ZIKV | Suckling C57BL/6, BALB/c and Kunming mice (multi-subcutaneous dorsal inoculation) | C57BL/6 owned the highest susceptibility and pathogenicity to the nervous system, referred to as movement disorders, with 100% incidence, while KM was characterized by lower limb weakness with 62% morbidity. |
| (Green et al., 2017) | WNV | Wild type and Oas1b-defective mice | Oligoadenylate-synthetase (Oas) impacts innate immune gene signatures that impact WNV infection. |
| (Daniels et al., 2017) | WNV | Ripk3^-/-^ and wild type mice (subcutaneous infection) | RIPK3 restricted WNV pathogenesis independently of cell death |
| (Hirsch et al., 2017) | ZIKV | Rhesus macaques (subcutaneous inoculation) | ZIKV was detected in peripheral nervous tissue and other organs at 7 days post-infection; viral RNA persisted in neuronal tissue 28-35 days post-infection. |
| (Tripathi et al., 2017) | ZIKV (African and Asian strains) | Stat2-/- mice (subcutaneous innoculation) | ZIKV spread to the central nervous system display neurological symptoms; African strains displayed higher neurovirulence than Asian strains |
| (Xavier-Neto et al., 2017) | ZIKV | Wild-type FVB/NJ and C57BL/6J mice (intravenous inoculation) | Early exposure to ZIKV produced complex manifestations of dysraphia and hydrocephalus. |
| (Vermillion et al., 2017) | ZIKV | Immunocompetent pregnant-mice (intrauterine inoculation) | Early intrauterine inoculation reduced neonatal brain cortical thickness and increased microglial activation |
| (van den Pol et al., 2017) | ZIKV | Neonatal mice (intraperitoneal inoculation) and IFNR-/- adult mice (intraocular inoculation) | Astrocytes were the first cells targeted after neonatal ZIKV-infection; neurons were affected later; ZIKV infected nucleus from the visual system. ZIKV infected glial cells from the visual system after intraocular inoculation. |
| (Smith et al., 2017) | ZIKV | C57BL/6 mice treated with an antibody to disrupt type I IFN signaling (subcutaneous and intraperitoneal inoculation | Acute to subacute encephalitis/encephalomyelitis that is characterized by neuronal death, astrogliosis, microgliosis, scattered necrotic cellular debris, and inflammatory cell infiltrates. |
| (Fernandes et al., 2017) | ZIKV | Neonatal Swiss mice (intracranial and subcutaneous inoculation) | Neuronal death, white matter degeneration and neutrophil infiltration were present in newborn mice inoculated by both routes; subcutaneous inoculation led to an important neurological manifestations, including myelopathy |
| (Dhole et al., 2016) | DENV-2 (P04/08) | IFN-a/b receptor knockout mice (intraperitoneal inoculation) | Virus initially grew in the non-neuronal organs with subsequent penetration into the central nervous system. |
| (Chan et al., 2016) | ZIKV | Dexamethasone-immunosupresed mice (intraperitoneal inoculation) | Mice developed disseminated infection, affecting the central nervous system; type I IFN treatment improved clinical outcomes |
| (Manangeeswaran et al., 2016) | ZIKV | Immunocompetent neonatal C57BL/6 and C57BL/6-IFNAR KO mice (subcutaneous inoculation) | Mice develop clinical neurological involvement; immunohistochemistry show viral antigen predominantly in cerebellum at the peak of the disease in both models. |
| (Goodfellow et al., 2016) | ZIKV | Chicken embryos (intra-amniotic inoculation) | ZIKV-infected embryos presented a microcephaly-like phenotype (enlarged ventricles and decreased cortical growth) |
| (Adams Waldorf et al., 2016) | ZIKV | Pregnant pigtail macaque (subcutaneous inoculation) | Pregnant macaque developed asymmetrical occipital-parietal lobe lesions; fetal brain revealed white matter hypoplasia, periventricular white matter gliosis and axonal and ependymal injury. |
| (Li et al., 2016c) | ZIKV | Rhesus monkey (subcutaneous inoculation) | ZIKV RNA was detected in brain, cerebellum and brainstem causing vascular cuffing |
| (Huang et al., 2016) | ZIKV | Neonatal C57BL/6 mice (intracranial inoculation) | ZIKV induced apoptosis throughout the brain and proliferating cells in the ventricular zones were depleted |
| (Maximova et al., 2016) | WNV | Rhesus monkeys (intracranial inoculation) | WNV infected neurons within the motor pathways; viral particles were found at the synaptic ends suggesting transsynaptic spread |
| (Yockey et al., 2016) | ZIKV | C57BL/6NCrl wild and IFNR-/- type (intravaginal inoculation) | Vaginal ZIKV infection in early pregnancy lead to fetal brain infection and growth restriction, which are more severe in IFNR-/- mice type |
| (Li et al., 2016b) | ZIKV | IRF-/- adult mice (intravenous inoculation) | ZIKV infected neural stem cells at the subventricular zone and the hippocampus, leading cell death and reduced proliferation |
| (Cugola et al., 2016) | ZIKV | SJL and C57BL/6 pregnant mice (intravenous inoculation) | ZIKV infected neurons in the fetal brain of the SJL model and caused apoptosis |
| (Li et al., 2016a) | ZIKV (Asian strain) | Embryonic mice (intracranial inoculation) | ZIKV targeted neural progenitor cells causing apoptosis, deceased replication, inhibition of differentiation |
| (Wu et al., 2016) | ZIKV | C57 pregnant mice (intraperitoneal inoculation) | ZIKV infected radial glial cells of dorsal ventricular zone of the fetuses and caused reduction of the cerebral cortex of the offspring |
| (de Souza et al., 2013) | DENV-3 | C57BL/6 and Nos2-/- type mice (intracranial inoculation) | DENV-3 neurovirulence is associated with a deleterious role of NOS2 in the brain |
| (Amorim et al., 2012) | DENV-2 (JHA1) | Immunocompetent adult Balb/c mice (intracerebral inoculation) | 100% lethality with the highest dose and 20% with the lowest; DENV infection caused extensive gliosis scars, neuronal death, mononuclear infiltrate in blood-brain-vessel; viable viral particles found in the brain. |
| (Velandia-Romero et al., 2012) | DENV-4 (adapted via brain passes) | Neonatal Balb/c mice (intraperitoneal inoculation) | Fatal encephalitis in mice infected 2-7 postnatal days (mice with later infection survived); manifestations included limb paralysis and instability; DENV RNA was found in microglial cells, neurons, oligodendrocytes and endothelial cells. |
| (Appler et al., 2010) | WNV | Adult mice (subcutaneous inoculation) | WNV persisted in the CNS and periphery of mice for up to 6 months post infection in mice with subclinical infection |
| (Shrestha et al., 2003) | WNV | C57Bl/6J mice (subcutaneous inoculation) | WNV infected neurons in the anterior horn of the spinal cord |
| (Despres et al., 1998) (Despres et al., 1996) | Various DENV strains | Neonatal mice (intracerebral inoculation) | Fatal encephalitis 10 days after the inoculation. DENV targeted cortex and hippocampal neurons, inducing apoptosis. |

Adams Waldorf, K.M., Stencel-Baerenwald, J.E., Kapur, R.P., Studholme, C., Boldenow, E., Vornhagen, J., et al. (2016). Fetal brain lesions after subcutaneous inoculation of Zika virus in a pregnant nonhuman primate. *Nat Med* 22(11)**,** 1256-1259. doi: 10.1038/nm.4193.

Amorim, J.H., Pereira Bizerra, R.S., dos Santos Alves, R.P., Sbrogio-Almeida, M.E., Levi, J.E., Capurro, M.L., et al. (2012). A genetic and pathologic study of a DENV2 clinical isolate capable of inducing encephalitis and hematological disturbances in immunocompetent mice. *PLoS One* 7(9)**,** e44984. doi: 10.1371/journal.pone.0044984.

Appler, K.K., Brown, A.N., Stewart, B.S., Behr, M.J., Demarest, V.L., Wong, S.J., et al. (2010). Persistence of West Nile virus in the central nervous system and periphery of mice. *PLoS One* 5(5)**,** e10649. doi: 10.1371/journal.pone.0010649.

Bortell, N., Flynn, C., Conti, B., Fox, H.S., and Marcondes, M.C.G. (2017). Osteopontin Impacts West Nile virus Pathogenesis and Resistance by Regulating Inflammasome Components and Cell Death in the Central Nervous System at Early Time Points. *Mediators Inflamm* 2017**,** 7582437. doi: 10.1155/2017/7582437.

Chan, J.F., Zhang, A.J., Chan, C.C., Yip, C.C., Mak, W.W., Zhu, H., et al. (2016). Zika Virus Infection in Dexamethasone-immunosuppressed Mice Demonstrating Disseminated Infection with Multi-organ Involvement Including Orchitis Effectively Treated by Recombinant Type I Interferons. *EBioMedicine* 14**,** 112-122. doi: 10.1016/j.ebiom.2016.11.017.

Cugola, F.R., Fernandes, I.R., Russo, F.B., Freitas, B.C., Dias, J.L., Guimaraes, K.P., et al. (2016). The Brazilian Zika virus strain causes birth defects in experimental models. *Nature* 534(7606)**,** 267-271. doi: 10.1038/nature18296.

Daniels, B.P., Snyder, A.G., Olsen, T.M., Orozco, S., Oguin, T.H., 3rd, Tait, S.W., et al. (2017). RIPK3 Restricts Viral Pathogenesis via Cell Death-Independent Neuroinflammation. *Cell* 169(2)**,** 301-313 e311. doi: 10.1016/j.cell.2017.03.011.

de Souza, K.P., Silva, E.G., de Oliveira Rocha, E.S., Figueiredo, L.B., de Almeida-Leite, C.M., Arantes, R.M., et al. (2013). Nitric oxide synthase expression correlates with death in an experimental mouse model of dengue with CNS involvement. *Virol J* 10**,** 267. doi: 10.1186/1743-422X-10-267.

Despres, P., Flamand, M., Ceccaldi, P.E., and Deubel, V. (1996). Human isolates of dengue type 1 virus induce apoptosis in mouse neuroblastoma cells. *J Virol* 70(6)**,** 4090-4096.

Despres, P., Frenkiel, M.P., Ceccaldi, P.E., Duarte Dos Santos, C., and Deubel, V. (1998). Apoptosis in the mouse central nervous system in response to infection with mouse-neurovirulent dengue viruses. *J Virol* 72(1)**,** 823-829.

Dhole, P., Nakayama, E.E., Saito, A., Limkittikul, K., Phanthanawiboon, S., Shioda, T., et al. (2016). Sequence diversity of dengue virus type 2 in brain and thymus of infected interferon receptor ko mice: implications for dengue virulence. *Virol J* 13(1)**,** 199. doi: 10.1186/s12985-016-0658-4.

Duggal, N.K., Ritter, J.M., McDonald, E.M., Romo, H., Guirakhoo, F., Davis, B.S., et al. (2017). Differential Neurovirulence of African and Asian Genotype Zika Virus Isolates in Outbred Immunocompetent Mice. *Am J Trop Med Hyg*. doi: 10.4269/ajtmh.17-0263.

Fernandes, N.C., Nogueira, J.S., Ressio, R.A., Cirqueira, C.S., Kimura, L.M., Fernandes, K.R., et al. (2017). Experimental Zika virus infection induces spinal cord injury and encephalitis in newborn Swiss mice. *Exp Toxicol Pathol* 69(2)**,** 63-71. doi: 10.1016/j.etp.2016.11.004.

Goodfellow, F.T., Tesla, B., Simchick, G., Zhao, Q., Hodge, T., Brindley, M.A., et al. (2016). Zika Virus Induced Mortality and Microcephaly in Chicken Embryos. *Stem Cells Dev* 25(22)**,** 1691-1697. doi: 10.1089/scd.2016.0231.

Green, R., Wilkins, C., Thomas, S., Sekine, A., Hendrick, D.M., Voss, K., et al. (2017). Oas1b-dependent Immune Transcriptional Profiles of West Nile Virus Infection in the Collaborative Cross. *G3 (Bethesda)* 7(6)**,** 1665-1682. doi: 10.1534/g3.117.041624.

Hirsch, A.J., Smith, J.L., Haese, N.N., Broeckel, R.M., Parkins, C.J., Kreklywich, C., et al. (2017). Zika Virus infection of rhesus macaques leads to viral persistence in multiple tissues. *PLoS Pathog* 13(3)**,** e1006219. doi: 10.1371/journal.ppat.1006219.

Ho, M.R., Tsai, T.T., Chen, C.L., Jhan, M.K., Tsai, C.C., Lee, Y.C., et al. (2017). Blockade of dengue virus infection and viral cytotoxicity in neuronal cells in vitro and in vivo by targeting endocytic pathways. *Sci Rep* 7(1)**,** 6910. doi: 10.1038/s41598-017-07023-z.

Huang, W.C., Abraham, R., Shim, B.S., Choe, H., and Page, D.T. (2016). Zika virus infection during the period of maximal brain growth causes microcephaly and corticospinal neuron apoptosis in wild type mice. *Sci Rep* 6**,** 34793. doi: 10.1038/srep34793.

Kuszpit, K., Hollidge, B.S., Zeng, X., Stafford, R.G., Daye, S., Zhang, X., et al. (2017). [18F]DPA-714 PET Imaging Reveals Global Neuroinflammation in Zika Virus-Infected Mice. *Mol Imaging Biol*. doi: 10.1007/s11307-017-1118-2.

Li, C., Xu, D., Ye, Q., Hong, S., Jiang, Y., Liu, X., et al. (2016a). Zika Virus Disrupts Neural Progenitor Development and Leads to Microcephaly in Mice. *Cell Stem Cell* 19(1)**,** 120-126. doi: 10.1016/j.stem.2016.04.017.

Li, H., Saucedo-Cuevas, L., Regla-Nava, J.A., Chai, G., Sheets, N., Tang, W., et al. (2016b). Zika Virus Infects Neural Progenitors in the Adult Mouse Brain and Alters Proliferation. *Cell Stem Cell* 19(5)**,** 593-598. doi: 10.1016/j.stem.2016.08.005.

Li, X.F., Dong, H.L., Huang, X.Y., Qiu, Y.F., Wang, H.J., Deng, Y.Q., et al. (2016c). Characterization of a 2016 Clinical Isolate of Zika Virus in Non-human Primates. *EBioMedicine* 12**,** 170-177. doi: 10.1016/j.ebiom.2016.09.022.

Manangeeswaran, M., Ireland, D.D., and Verthelyi, D. (2016). Zika (PRVABC59) Infection Is Associated with T cell Infiltration and Neurodegeneration in CNS of Immunocompetent Neonatal C57Bl/6 Mice. *PLoS Pathog* 12(11)**,** e1006004. doi: 10.1371/journal.ppat.1006004.

Maximova, O.A., Bernbaum, J.G., and Pletnev, A.G. (2016). West Nile Virus Spreads Transsynaptically within the Pathways of Motor Control: Anatomical and Ultrastructural Mapping of Neuronal Virus Infection in the Primate Central Nervous System. *PLoS Negl Trop Dis* 10(9)**,** e0004980. doi: 10.1371/journal.pntd.0004980.

Oh, Y., Zhang, F., Wang, Y., Lee, E.M., Choi, I.Y., Lim, H., et al. (2017). Zika virus directly infects peripheral neurons and induces cell death. *Nat Neurosci* 20(9)**,** 1209-1212. doi: 10.1038/nn.4612.

Paul, A.M., Acharya, D., Duty, L., Thompson, E.A., Le, L., Stokic, D.S., et al. (2017). Osteopontin facilitates West Nile virus neuroinvasion via neutrophil "Trojan horse" transport. *Sci Rep* 7(1)**,** 4722. doi: 10.1038/s41598-017-04839-7.

Shao, Q., Herrlinger, S., Zhu, Y.N., Yang, M., Goodfellow, F., Stice, S.L., et al. (2017). The African Zika virus MR-766 is more virulent and causes more severe brain damage than current Asian lineage and Dengue virus. *Development*. doi: 10.1242/dev.156752.

Shrestha, B., Gottlieb, D., and Diamond, M.S. (2003). Infection and injury of neurons by West Nile encephalitis virus. *J Virol* 77(24)**,** 13203-13213.

Smith, D.R., Hollidge, B., Daye, S., Zeng, X., Blancett, C., Kuszpit, K., et al. (2017). Neuropathogenesis of Zika Virus in a Highly Susceptible Immunocompetent Mouse Model after Antibody Blockade of Type I Interferon. *PLoS Negl Trop Dis* 11(1)**,** e0005296. doi: 10.1371/journal.pntd.0005296.

Tripathi, S., Balasubramaniam, V.R., Brown, J.A., Mena, I., Grant, A., Bardina, S.V., et al. (2017). A novel Zika virus mouse model reveals strain specific differences in virus pathogenesis and host inflammatory immune responses. *PLoS Pathog* 13(3)**,** e1006258. doi: 10.1371/journal.ppat.1006258.

van den Pol, A.N., Mao, G., Yang, Y., Ornaghi, S., and Davis, J.N. (2017). Zika Virus Targeting in the Developing Brain. *J Neurosci* 37(8)**,** 2161-2175. doi: 10.1523/JNEUROSCI.3124-16.2017.

Velandia-Romero, M.L., Acosta-Losada, O., and Castellanos, J.E. (2012). In vivo infection by a neuroinvasive neurovirulent dengue virus. *J Neurovirol* 18(5)**,** 374-387. doi: 10.1007/s13365-012-0117-y.

Vermillion, M.S., Lei, J., Shabi, Y., Baxter, V.K., Crilly, N.P., McLane, M., et al. (2017). Intrauterine Zika virus infection of pregnant immunocompetent mice models transplacental transmission and adverse perinatal outcomes. *Nat Commun* 8**,** 14575. doi: 10.1038/ncomms14575.

Wang, Z.Y., Wang, Z., Zhen, Z.D., Feng, K.H., Guo, J., Gao, N., et al. (2017). Axl is not an indispensable factor for Zika virus infection in mice. *J Gen Virol* 98(8)**,** 2061-2068. doi: 10.1099/jgv.0.000886.

Wu, K.Y., Zuo, G.L., Li, X.F., Ye, Q., Deng, Y.Q., Huang, X.Y., et al. (2016). Vertical transmission of Zika virus targeting the radial glial cells affects cortex development of offspring mice. *Cell Res* 26(6)**,** 645-654. doi: 10.1038/cr.2016.58.

Xavier-Neto, J., Carvalho, M., Pascoalino, B.D., Cardoso, A.C., Costa, A.M., Pereira, A.H., et al. (2017). Hydrocephalus and arthrogryposis in an immunocompetent mouse model of ZIKA teratogeny: A developmental study. *PLoS Negl Trop Dis* 11(2)**,** e0005363. doi: 10.1371/journal.pntd.0005363.

Yockey, L.J., Varela, L., Rakib, T., Khoury-Hanold, W., Fink, S.L., Stutz, B., et al. (2016). Vaginal Exposure to Zika Virus during Pregnancy Leads to Fetal Brain Infection. *Cell* 166(5)**,** 1247-1256 e1244. doi: 10.1016/j.cell.2016.08.004.

Yoon, K.J., Song, G., Qian, X., Pan, J., Xu, D., Rho, H.S., et al. (2017). Zika-Virus-Encoded NS2A Disrupts Mammalian Cortical Neurogenesis by Degrading Adherens Junction Proteins. *Cell Stem Cell* 21(3)**,** 349-358 e346. doi: 10.1016/j.stem.2017.07.014.

Yu, J., Liu, X., Ke, C., Wu, Q., Lu, W., Qin, Z., et al. (2017). Effective Suckling C57BL/6, Kunming, and BALB/c Mouse Models with Remarkable Neurological Manifestation for Zika Virus Infection. *Viruses* 9(7). doi: 10.3390/v9070165.
