# Supplementary material for: In vitro and in silico Models to Study Mosquito-Borne Flavivirus Neuropathogenesis, Prevention, and Treatment
Source: Front Cell Infect Microbiol. 2019 Jul 9;9:223. doi: 10.3389/fcimb.2019.00223 (PMC6629778; doi:10.3389/fcimb.2019.00223)
Supplement: Supplementary file 3 [file Table_3.DOCX]

**Table 3. Animal models: neuropathogenesis/immunology**

| Author | Virus type | Model | Findings |
| --- | --- | --- | --- |
| (Bardina et al., 2017) | WNV | Ccr7-deficient and wild type mice | Ccr7-deficient mice had significantly higher CNS viral loads and mortality rates than wild-type animals; Ccr7 was s a critical host defense restriction factor limiting neuroinflammation during acute viral infection |
| (Al-Shujairi et al., 2017) | DENV-2 | Immunocompetent C57BL/6 mice (intracranial inoculation) | DENV-2 brain infection induced interferon and T-cell (CD8^+^) infiltrative response. |
| (Graham et al., 2016) | WNV | Recombinant inbred mouse (subcutaneous inoculation) | Persistent WNV loads in the brain; regulatory T cell response restrained the immune response facilitating chronic infection |
| (Oliveira et al., 2016) (Merge with the other Oliveira) | DENV-2 (neuroadapted) | Immunocompetent adult BALB/c mice (intracerebral inoculation) | Cellular and humoral response in the CNS and peripherally, T-cell lymphocytic infiltration in the brain |
| (Vasconcelos et al., 2016) | DENV-3 and DENV-2 | Non-human primate (Black-penicilled Marmoset) (subcutaneous inoculation) | Marked morphological changes in microglial population associated with intense TNFq positive immunolabeling |
| (Graham et al., 2015) | WNV | Collaborative Cross mice (multiple strains) | The CC effectively modeled a wide range of WNV clinical, virologic, and immune phenotypes, |
| (Durrant et al., 2014) | WNV | C57BL/6 wild and IL-1R1-/- type mice (subcutaneous inoculation) | IL-1 promoted T-cell adhesion to brain endothelial cells in WNV infection with no alteration in the permeability of the BBB |
| (Roe et al., 2014) | WNV | C57BL/6 mice (subcutaneous inoculation) | WNV infection induced ICAM-1, VCAM-1 and E-selectin; the BBB permeability increased following the migration of monocytes and lymphocytes |
| (Sabouri et al., 2014) | WNV | MyD88/TRIF-/- mice (subcutaneous inoculation) | Lack of TLR adapter molecules increased susceptibility to WNV infection and impaired innate immunity |
| (Clarke et al., 2014) | WNV | Immunocompetent adult mice (intracranial inoculation) | WNV induced neuronal injury is mediated by death receptors-induced apoptosis signaling, even in absence of immune cells |
| (Kumar et al., 2012) | WNV | C57BL/6 and db/db diabetic type mice | Increased WNV replication observed in brain of db/db mice, enhanced inflammatory response and delayed induction of antiviral response |
| (Falconar and Martinez, 2011) | DENV-2 strains | TO mice (immunization with different purified NS antigens) | Mice developed meningo-encephalitis, with greater than 90,000-fold DENV-2 AER titers in microglial cells located throughout their brain parenchyma, some of which formed nodules around dead neurons. |
| (Lim et al., 2011) | WNV | C57BL/6 wild and Ccr2-/- type mice | Deficiency of Ccr2 increased mortality due to encephalitis and decreased monocyte (Ly6c) accumulation in the brain |
| (Zhang et al., 2010) | WNV | Adult mice (subcutaneous inoculation) | TNF-alpha expression during WNV encephalitis may be an adaptive response to diminish CXCL10-induced death |
| (Brien et al., 2009) | WNV | Old mice (intraperitoneal and subcutaneous inoculation) | Old mice had increased susceptibility to WNV brain infection due to defects in T CD8 and CD4 cells |
| (Garcia-Tapia et al., 2007) | WNV | C57Bl/6 mice (subcutaneous inoculation) | Chemokines MCP-5, IP-10, and MIG triggered early inflammation in brain, followed by IFN-g and TNF-a expression |

Al-Shujairi, W.H., Clarke, J.N., Davies, L.T., Alsharifi, M., Pitson, S.M., and Carr, J.M. (2017). Intracranial Injection of Dengue Virus Induces Interferon Stimulated Genes and CD8+ T Cell Infiltration by Sphingosine Kinase 1 Independent Pathways. *PLoS One* 12(1)**,** e0169814. doi: 10.1371/journal.pone.0169814.

Bardina, S.V., Brown, J.A., Michlmayr, D., Hoffman, K.W., Sum, J., Pletnev, A.G., et al. (2017). Chemokine Receptor Ccr7 Restricts Fatal West Nile Virus Encephalitis. *J Virol* 91(10). doi: 10.1128/JVI.02409-16.

Brien, J.D., Uhrlaub, J.L., Hirsch, A., Wiley, C.A., and Nikolich-Zugich, J. (2009). Key role of T cell defects in age-related vulnerability to West Nile virus. *J Exp Med* 206(12)**,** 2735-2745. doi: 10.1084/jem.20090222.

Clarke, P., Leser, J.S., Quick, E.D., Dionne, K.R., Beckham, J.D., and Tyler, K.L. (2014). Death receptor-mediated apoptotic signaling is activated in the brain following infection with West Nile virus in the absence of a peripheral immune response. *J Virol* 88(2)**,** 1080-1089. doi: 10.1128/JVI.02944-13.

Durrant, D.M., Daniels, B.P., and Klein, R.S. (2014). IL-1R1 signaling regulates CXCL12-mediated T cell localization and fate within the central nervous system during West Nile Virus encephalitis. *J Immunol* 193(8)**,** 4095-4106. doi: 10.4049/jimmunol.1401192.

Falconar, A.K., and Martinez, F. (2011). The NS1 glycoprotein can generate dramatic antibody-enhanced dengue viral replication in normal out-bred mice resulting in lethal multi-organ disease. *PLoS One* 6(6)**,** e21024. doi: 10.1371/journal.pone.0021024.

Garcia-Tapia, D., Hassett, D.E., Mitchell, W.J., Jr., Johnson, G.C., and Kleiboeker, S.B. (2007). West Nile virus encephalitis: sequential histopathological and immunological events in a murine model of infection. *J Neurovirol* 13(2)**,** 130-138. doi: 10.1080/13550280601187185.

Graham, J.B., Swarts, J.L., Wilkins, C., Thomas, S., Green, R., Sekine, A., et al. (2016). A Mouse Model of Chronic West Nile Virus Disease. *PLoS Pathog* 12(11)**,** e1005996. doi: 10.1371/journal.ppat.1005996.

Graham, J.B., Thomas, S., Swarts, J., McMillan, A.A., Ferris, M.T., Suthar, M.S., et al. (2015). Genetic diversity in the collaborative cross model recapitulates human West Nile virus disease outcomes. *MBio* 6(3)**,** e00493-00415. doi: 10.1128/mBio.00493-15.

Kumar, M., Roe, K., Nerurkar, P.V., Namekar, M., Orillo, B., Verma, S., et al. (2012). Impaired virus clearance, compromised immune response and increased mortality in type 2 diabetic mice infected with West Nile virus. *PLoS One* 7(8)**,** e44682. doi: 10.1371/journal.pone.0044682.

Lim, J.K., Obara, C.J., Rivollier, A., Pletnev, A.G., Kelsall, B.L., and Murphy, P.M. (2011). Chemokine receptor Ccr2 is critical for monocyte accumulation and survival in West Nile virus encephalitis. *J Immunol* 186(1)**,** 471-478. doi: 10.4049/jimmunol.1003003.

Oliveira, E.R., Amorim, J.F., Paes, M.V., Azevedo, A.S., Goncalves, A.J., Costa, S.M., et al. (2016). Peripheral effects induced in BALB/c mice infected with DENV by the intracerebral route. *Virology* 489**,** 95-107. doi: 10.1016/j.virol.2015.12.006.

Roe, K., Orillo, B., and Verma, S. (2014). West Nile virus-induced cell adhesion molecules on human brain microvascular endothelial cells regulate leukocyte adhesion and modulate permeability of the in vitro blood-brain barrier model. *PLoS One* 9(7)**,** e102598. doi: 10.1371/journal.pone.0102598.

Sabouri, A.H., Marcondes, M.C., Flynn, C., Berger, M., Xiao, N., Fox, H.S., et al. (2014). TLR signaling controls lethal encephalitis in WNV-infected brain. *Brain Res* 1574**,** 84-95. doi: 10.1016/j.brainres.2014.05.049.

Vasconcelos, B.C., Vieira, J.A., Silva, G.O., Fernandes, T.N., Rocha, L.C., Viana, A.P., et al. (2016). Antibody-enhanced dengue disease generates a marked CNS inflammatory response in the black-tufted marmoset Callithrix penicillata. *Neuropathology* 36(1)**,** 3-16. doi: 10.1111/neup.12229.

Zhang, B., Patel, J., Croyle, M., Diamond, M.S., and Klein, R.S. (2010). TNF-alpha-dependent regulation of CXCR3 expression modulates neuronal survival during West Nile virus encephalitis. *J Neuroimmunol* 224(1-2)**,** 28-38. doi: 10.1016/j.jneuroim.2010.05.003.
