# Supplementary material for: In vitro and in silico Models to Study Mosquito-Borne Flavivirus Neuropathogenesis, Prevention, and Treatment
Source: Front Cell Infect Microbiol. 2019 Jul 9;9:223. doi: 10.3389/fcimb.2019.00223 (PMC6629778; doi:10.3389/fcimb.2019.00223)
Supplement: Supplementary file 4 [file Table_4.DOCX]

**Table 4. Animal models: vaccines**

| Author | Virus type | Model | Vaccine | Findings |
| --- | --- | --- | --- | --- |
| (Wong et al., 2017) | DENV | Non-human primate model (intraperitoneal infection) | Vaccine (CYD-TDV) | After immunization, no DENV RNA or histopathological changes were detected in the brain (no BBB cross). |
| (Zheng et al., 2017) | DENV | BALB/c mice (intramuscular injection) | Vaccine (DNA against DENV-1 and DENV-2 (pVAX-D1ME/F2ME)) | Vaccine induced persistent humoral and cellular responses and effectively protected mice against DENV-1 and DENV-2 lethal challenge. |
| (Valdes et al., 2017) | DENV | BALB/c mice (intraperitoneal inoculation) | Vaccine (tetravalent DIIIC) | The Tetra DIIIC formulation induced a humoral immune response against the four DENV serotypes |
| (Chahal et al., 2017) | ZIKV | C57BL/6 mice (intramuscular injection) | Vaccine (RNA nanoparticle) | RNA nanoparticle vaccine elicited ZIKV E protein-specific IgG and CD8 responses in C57BL/6 mice |
| (Oliveira et al., 2016) | DENV (pcTPANS1 – DNA vaccine NS1) | Immunocompetent mice (intracerebral inoculation) | Vaccine/ pathophysiology | T-cell activation in spleen; migration of activated T-cells |
| (Keelapang et al., 2013) | DENV-1/2 | BALB/c adult mice and rhesus macaques (Macaca mulatta) (subcutaneous inoculation) | Vaccine ((prM+E) chimeric live attenuated vaccine with enhanced pRM cleavage) | Vaccine induced neutralizing antibody response in mice and rhesus macaques |

Chahal, J.S., Fang, T., Woodham, A.W., Khan, O.F., Ling, J., Anderson, D.G., et al. (2017). An RNA nanoparticle vaccine against Zika virus elicits antibody and CD8+ T cell responses in a mouse model. *Sci Rep* 7(1)**,** 252. doi: 10.1038/s41598-017-00193-w.

Keelapang, P., Nitatpattana, N., Suphatrakul, A., Punyahathaikul, S., Sriburi, R., Pulmanausahakul, R., et al. (2013). Generation and preclinical evaluation of a DENV-1/2 prM+E chimeric live attenuated vaccine candidate with enhanced prM cleavage. *Vaccine* 31(44)**,** 5134-5140. doi: 10.1016/j.vaccine.2013.08.027.

Oliveira, E.R., Goncalves, A.J., Costa, S.M., Azevedo, A.S., Mantuano-Barradas, M., Nogueira, A.C., et al. (2016). Aspects of T Cell-Mediated Immunity Induced in Mice by a DNA Vaccine Based on the Dengue-NS1 Antigen after Challenge by the Intracerebral Route. *PLoS One* 11(9)**,** e0163240. doi: 10.1371/journal.pone.0163240.

Valdes, I., Marcos, E., Suzarte, E., Perez, Y., Brown, E., Lazo, L., et al. (2017). A dose-response study in mice of a tetravalent vaccine candidate composed of domain III-capsid proteins from dengue viruses. *Arch Virol* 162(8)**,** 2247-2256. doi: 10.1007/s00705-017-3360-y.

Wong, G., He, S., Siragam, V., Bi, Y., Mbikay, M., Chretien, M., et al. (2017). Antiviral activity of quercetin-3-beta-O-D-glucoside against Zika virus infection. *Virol Sin*. doi: 10.1007/s12250-017-4057-9.

Zheng, X., Chen, H., Wang, R., Fan, D., Feng, K., Gao, N., et al. (2017). Effective Protection Induced by a Monovalent DNA Vaccine against Dengue Virus (DV) Serotype 1 and a Bivalent DNA Vaccine against DV1 and DV2 in Mice. *Front Cell Infect Microbiol* 7**,** 175. doi: 10.3389/fcimb.2017.00175.
