# Supplementary material for: In vitro and in silico Models to Study Mosquito-Borne Flavivirus Neuropathogenesis, Prevention, and Treatment
Source: Front Cell Infect Microbiol. 2019 Jul 9;9:223. doi: 10.3389/fcimb.2019.00223 (PMC6629778; doi:10.3389/fcimb.2019.00223)
Supplement: Supplementary file 5 [file Table_5.DOCX]

**Table 5. Animal models: drugs**

| Author | Virus type | Model | Medication | Findings |
| --- | --- | --- | --- | --- |
| (Li et al., 2017) | ZIKV | Embryonic mice (transplacentally) | Drug (chloroquine) | Chloroquine protects embryonic brains from ZIKV infection and microcephaly |
| (Stein et al., 2017) | ZIKV | Immunocompromised C57BL/6 (IFN-aR1-/-) mice (intraperitoneal infection) | Drug (human polyclonal antibodies after cattle vaccination (ZIKV DNA vaccine)) | Antibody treatment eliminated ZIKV induced tissue damage in brain and testes. |
| (Ferreira et al., 2017) | ZIKV | Neonatal Swiss mice | Drug (Sofosbuvir) | Sofosbuvir reduced the levels of ZIKV from 60%-90% in plasma, spleen, kidney and brain; prevented neuromotor impairment and loss of hippocampal and amygdala dependent memory. |
| (Costa et al., 2017) | ZIKV | Immunocompromise mice (IFN-aR1-/-) (intravenous inoculation) | Drug (NDMA receptor blockade) | Memantine massively reduced neurodegeneration and microgliosis in the brain of infected mice. |
| (Hu et al., 2016) | DENV-2 | Adult Balb/c mice (intracerebral inoculation) | Antiviral drug (liposome encapsulated LE-PolyICLC) | Reduction of viral titers and expression of viral E protein in the brain |
| (Lazear et al., 2015) | WNV | Wild and IFNR-/- type mice (intracerebral inoculation) | Antiviral (pegylated interferon) | Treatment of mice with pegylated interferon-λ2 resulted in decreased blood-brain barrier permeability, reduced West Nile virus infection in the brain without affecting viremia, and improved survival against lethal virus challenge |
| (Srivastava et al., 2015) | WNV | BALB/c mice (intraperitoneal inoculation) | Antiviral drug (WNV-IVIg) | WNV-IVIg inhibited lethal encephalitis by suppressing infiltration of CD45+ leukocytes and monocytes into the CNS |
| (Hunsperger and Roehrig, 2009) | WNV | C57BL/6J (subcutaneous inoculation) | Drug (Nocodazole) | Nocodazole delayed but not blocked WNV infection in the brain |

Costa, V.V., Del Sarto, J.L., Rocha, R.F., Silva, F.R., Doria, J.G., Olmo, I.G., et al. (2017). N-Methyl-d-Aspartate (NMDA) Receptor Blockade Prevents Neuronal Death Induced by Zika Virus Infection. *MBio* 8(2). doi: 10.1128/mBio.00350-17.

Ferreira, A.C., Zaverucha-do-Valle, C., Reis, P.A., Barbosa-Lima, G., Vieira, Y.R., Mattos, M., et al. (2017). Sofosbuvir protects Zika virus-infected mice from mortality, preventing short- and long-term sequelae. *Sci Rep* 7(1)**,** 9409. doi: 10.1038/s41598-017-09797-8.

Hu, Y., Hu, Y., Sun, L., Wong, J., and Wang, M. (2016). Antiviral effects of liposome-encapsulated PolyICLC against Dengue virus in a mouse model. *Biochem Biophys Res Commun* 478(2)**,** 913-918. doi: 10.1016/j.bbrc.2016.08.050.

Hunsperger, E.A., and Roehrig, J.T. (2009). Nocodazole delays viral entry into the brain following footpad inoculation with West Nile virus in mice. *J Neurovirol* 15(3)**,** 211-218. doi: 10.1080/13550280902913255.

Lazear, H.M., Daniels, B.P., Pinto, A.K., Huang, A.C., Vick, S.C., Doyle, S.E., et al. (2015). Interferon-lambda restricts West Nile virus neuroinvasion by tightening the blood-brain barrier. *Sci Transl Med* 7(284)**,** 284ra259. doi: 10.1126/scitranslmed.aaa4304.

Li, C., Zhu, X., Ji, X., Quanquin, N., Deng, Y.Q., Tian, M., et al. (2017). Chloroquine, a FDA-approved Drug, Prevents Zika Virus Infection and its Associated Congenital Microcephaly in Mice. *EBioMedicine* 24**,** 189-194. doi: 10.1016/j.ebiom.2017.09.034.

Srivastava, R., Ramakrishna, C., and Cantin, E. (2015). Anti-inflammatory activity of intravenous immunoglobulins protects against West Nile virus encephalitis. *J Gen Virol* 96(Pt 6)**,** 1347-1357. doi: 10.1099/vir.0.000079.

Stein, D.R., Golden, J.W., Griffin, B.D., Warner, B.M., Ranadheera, C., Scharikow, L., et al. (2017). Human polyclonal antibodies produced in transchromosomal cattle prevent lethal Zika virus infection and testicular atrophy in mice. *Antiviral Res* 146**,** 164-173. doi: 10.1016/j.antiviral.2017.09.005.
